# Response analysis of host *Spodoptera exigua* larvae to infection by Heliothis virescens ascovirus 3h (HvAV-3h) via transcriptome

**DOI:** 10.1038/s41598-018-23715-6

**Published:** 2018-03-29

**Authors:** Huan Yu, Zi-Qi Li, Lei He, Yi-Yi Ou-Yang, Ni Li, Guo-Hua Huang

**Affiliations:** 1 Hunan Provincial Key Laboratory for Biology and Control of Plant Diseases and Insect Pests, Hunan Agricultural University, Changsha, Hunan 410128 P. R. China; 2 College of Plant Protection, Hunan Agricultural University, Changsha, Hunan 410128 P. R. China

## Abstract

Heliothis virescens ascovirus 3 h (HvAV-3h), a dsDNA insect virus, belonging to the family *Ascoviridae*, can infect caterpillars of several Noctuidae species by ovipositing parasitoid wasps. In order to provide a comprehensive overview of the interactive responses of host larvae after infection by the ascovirus, a transcriptome analysis of *Spodoptera exigua* to HvAV-3h was conducted from 6 to 168 hours post infection (hpi). Approximately 101.64 Gb of RNA sequencing (RNA-seq) data obtained from infected and uninfected *S. exigua* larvae were used to perform a *de novo* transcriptome assembly, which generated approximately 62,258 *S. exigua* unigenes. Using differential gene expression analysis, it was determined that the majority of host transcripts were down-regulated beginning at 6 hpi and continuing throughout the infection period, although there was an increase in up-regulated unigene number during the 12 to 72 hpi stage. It is noteworthy that the most abundantly enriched pathways in KEGG annotation were Metabolism terms, indicating that the host larval metabolic mechanisms were highly influenced post HvAV-3h infection. In addition, the host cuticle protein encoding unigenes were highly down-regulated in most of the situations, suggesting that the host larval cuticle synthesis were inhibited by the viral infection.

## Introduction

After long periods of evolution, different kinds of complexity battles or cooperative strategies between microorganisms and their hosts present at all times. As a relatively unknown family of insect virus species, ascovirids are double-stranded DNA insect viruses with a genome size ranging from 100 to 200 kilo bp^1–7^, with a unique life cycle involving both the lepidopterous host larvae and hymenopterous parasitic wasp carrier^8–10^. A typical symptom that characterizes the infection of ascoviruses in host larvae is the change of color in the hemolymph into a milky white appearance^2^. The numerous vesicles with rod-shaped virions found in the affected hemolymph are thought to be responsible for the color change^2,11^. The first ascovirus strain was found in France in 1983^3^, with the family *Ascoviridae* being established by the International Committee of Virus Taxonomy (ICTV) in 1999. The poor *per os* infectivity and rarely manifested infections are probably the main reasons for the late discovery of ascoviruses^2,12,13^.

Heliothis virescens ascovirus 3 h (HvAV-3h), which was the first ascovirus isolate reported from China^7^, shares a 90% homologous identity to the virus HvAV-3e and 80% homologous identity to SfAV-1a^4^. Spodoptera frugiperda ascovirus 1a (SfAV-1a) was the first isolated ascovirus strain and was the type specie of ascovirus^3^. Compared to SfAV-1a, Heliothis virescens ascovirus 3e (HvAV-3e) was a relatively broad host range ascovirus strain^8^. The isolate HvAV-3h can be transmitted by the braconid wasp, *Microplitis similis*^9^ and lead to a 94.4 ± 3.7%, 88.9 ± 3.7%, and 94.4 ± 1.5% mortality in the noctuid species, *Helicoverpa armigera, Spodopera exigua*, and *S. litura*, respectively^14^. As a high virulence ascovirus isolate, HvAV-3h infection leads to a physical growth inhibition in the host larvae, with an extended larval developmental period (5–20 additional developmental days required by the *S. litura, S. exigua, and H. armigera* larvae)^14^. Although many virus infections are known to slow down or retard the growth of their host larvae, it was interesting to note the markedly prolonged life-spans that were observed in host larvae infected with ascoviruses. For example, the well-characterized ecdysteroid UDP-glucosyltransferase (EGT) protein encoded by baculoviruses inactivates ecdysteroids by sugar conjugation in infected host larvae. This glycosylation often leads to failure to molt in baculovirus infected insects. As a consequence, they continue to eat and grow during the time that they would normally be in the premolt wandering phase^15,16^. This is thought to provide an advantage for the baculovirus, as the larger larvae produce more progeny virus. Another example of viruses interfering with host larval growth is when viruses block the host cell cycles to remain in a certain phase, or they can inhibit the host cell apopotosis to gain sufficient time to complete their own replication and assembling.

To defend against viral infections, host larvae activate an abundance of immune responses to attack the viruses and protect their healthy tissues. These defensive reactions can be divided into several stages according to the virus infection phase. Using baculovirus as an example, the viral infection is mainly divided into four phases: immediate early, early, late, and very late. Different major issues occur in different infection phases, resulting in different immune defense reactions being expressed by the host. As the first lepidopteran insect to be genome sequenced, *Bombyx mori* can be used to illustrate the global host genes in response to different conditions. It has been reported that, when infected with the virus *Bombyx mori* nucleopolyhedrovirus (BmNPV), seven up-regulated and four down-regulated genes were identified by subtractive hybridization and Northern blot analysis^17^. Suppression subtractive hybridization (SSH) studies have shown that a hundred genes may be involved in the silkworm antiviral mechanism^18,19^. As transcriptomes have been widely used in such studies, host genes in different sets including the cytoskeleton, transcription, translation, energy metabolism, ubiquitin-proteasome, apoptosis and other pathways were identified and classified to provide a comprehensive view of host reaction in different stages.

Abundant research involving baculovirus and their host larval interactions based on transcriptome analysis have been conducted^20–24^. Because Ascoviridae is still a relatively recently characterized insect virus family, transcriptome analyses have not yet to be reported in investigations involving host reactions during ascovirus infections. In order to identify the diverse responses instigated by HvAV-3h infection, larvae of the natural host, *S. exigua*, were used to sequence the total mRNA via Illumaina sequencing technology. The results obtained in this study would provide a global view of molecular changes in *S. exigua* larvae during HvAV-3h infection, and may provide some reliable evidence for further studies on interactions between HvAV-3h and *S. exigua* larvae.

## Results

### Assembly and annotation of the *S. exigua* larvae transcriptome

To analyze the larval response to HvAV-3h infection, the 3rd instar *S. exigua* larvae were infected with HvAV-3h, and messenger RNAs were extracted for transcriptome analysis at 6, 12, 72 and 168 hpi. Healthy larvae were used as control (CK). Approximately 101.64 Gb of clean data were obtained after removing viral reads (approximately 3.39% of the total reads, Table S1), rRNA, and low quality reads from the raw data. The resulting high quality reads were subsequently assembled into 62,258 unigenes, with a total length of 116,089,209 bp, a mean length of 1,864 bp, an N50 value of 4,062 bp, and a GC% value of 41.50% (Table S2). A sequence similarity search identified 38,510 unigenes (61.9%) similarities to existing GenBank entries. These annotated unigenes showed significant similarity to sequences from various insect species, in which 44.1% unigenes were similar to *Bombyx mori*, 24.46% unigenes were similar to *Danaus plexippus*, 1.61% unigenes were similar to *Papilio xuthus*, 1.52% unigenes were similar to *Tribolium castaneum*, and 28.32% unigenes were similar to other species according to annotations in the UniProt Protein database (Table S3). The unigenes were further used to annotate with seven databases (NR, NT, SwissProt, COG, KEGG, GO, and InterPro). In summary, 36,453 unigenes were NR-annotated (58.55%), 33,582 unigenes were NT-annotated (53.94%), 29,490 unigenes were SwissPort-annotated (47.37%), 29,458 unigenes were KEGG-annotated (47.32%), 17,452 unigenes were COG-annotated (27.03%), 28,697 unigenes were InterPro-annotated (46.09%), and 6,767 unigenes were GO-annotated (10.87%). Overall, 43,723 unigenes were annotated with the seven databases. There were 37428 unigenes annotated with NR, InterPro, SwissProt, COG and KEGG, and 14,997 unigenes annotated by all five databases (Fig. S1) (Table S4).

### General *S. exigua* larval responses to HvAV-3h infection

To determine the expression of *S. exigua* larval unigenes and HvAV-3h ORFs at different time points post infection, the screened RNA-seq reads from each time point of the three replicated groups were mapped to the 62,258 assembled unigenes. Finally, a total of 62,258 host unigenes were at least 1 fragment mapping in 3 out of 15 samples. Gene expression levels were calculated as FPKM values. Fragment counts and average FPKM values for each unigene are provided in Tables S5 and S6, respectively, in the supplemental material.

**Figure 1:**
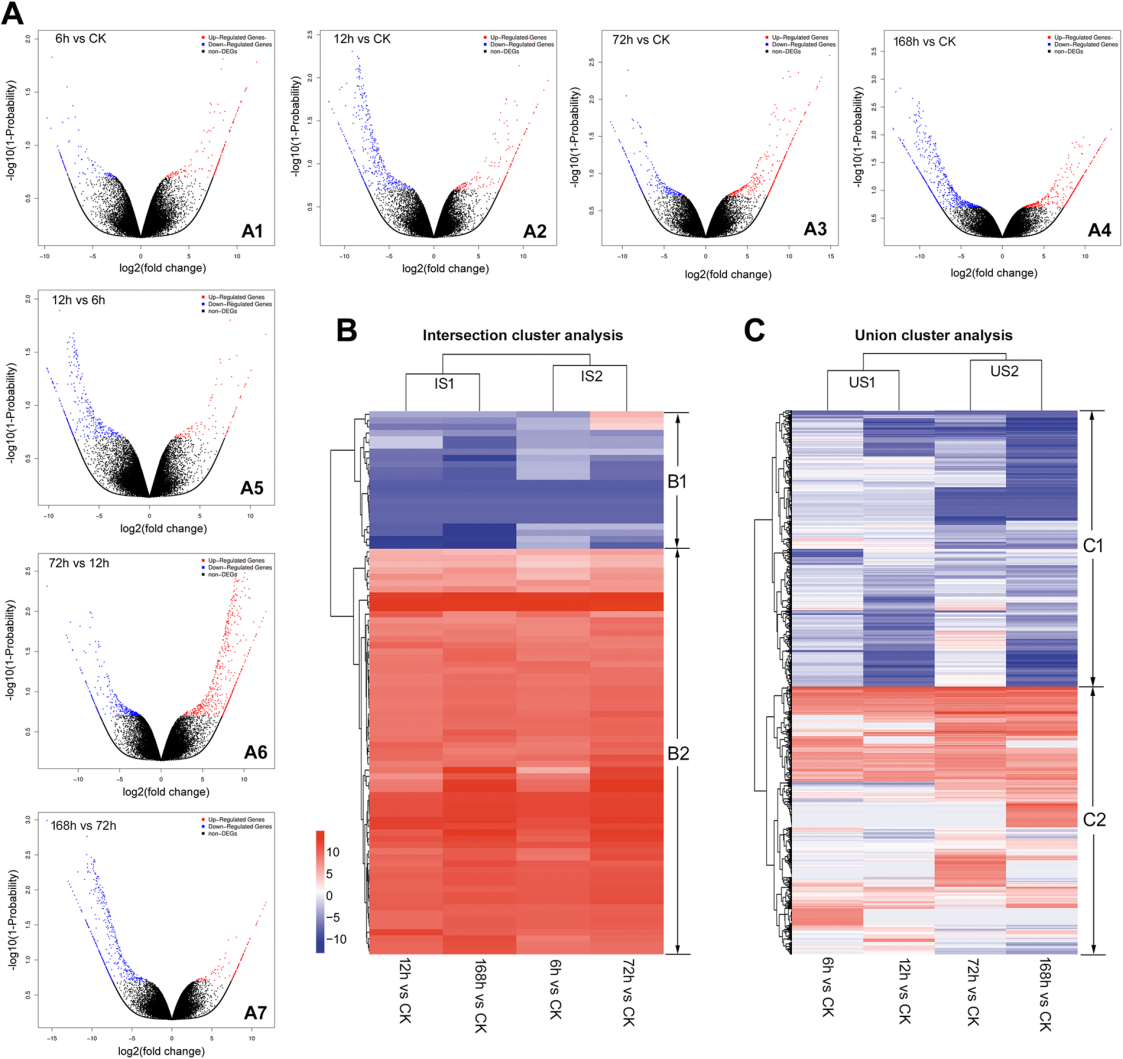
General analyses of host larval expressed genes responding to HvAV-3h infection. (**A**) The volcano plots of up-regulated unigenes (Red dots), down-regulated unigenes (Blue dots), and non-differentially expressed unigenes (Black dots) illustrating comparisons between different time points versus CK (the horizontal axis, A1 to A4) and comparisons between adjacent time points (the vertical axis, A1, A5 to A7). Cluster analyses of DEGs from different HvAV-3h infected time points compared with CK. In the intersection cluster analysis of DEGs (**B**), two major sub-groups were included (B1-B2), the DEGs from 12 h vs CK showed a expression pattern similar to 168 h vs CK (IS1), the remaining two compared groups were clustered into an IS2 group. In the union DEGs cluster analysis (**C**), two major sub-groups were included (C1-C2), the DEGs from 6 h vs CK showed a expression pattern similar to 12 h vs CK (US1), the remaining two compared group were clustered into a US2 sub-group.

To identify significantly up- or down-regulated unigenes, we selected only those unigenes with fold change levels ≥2 with a probability ≥0.8 within each comparison. The total number of DEGs (up-regulated or down-regulated) at different time points post infection are shown in Fig. S2. To analyze how viral infection caused changes in unigene expression, the expression level of each unigene during each time point post infection was compared with that of the same unigene at mock or at an adjacent infection time point (Fig. 1A1–4, a parallel time point). The comparisons of adjacent infection times (vertical axis) are also shown in Fig. 1A1 and A5–7. During comparison of adjacent infection times, the number of host larval up-regulated genes increased substantially from 12 to 72 hpi, nearly 1.2–2.4 fold of up-regulated genes more than other adjacent infection comparisons. To better understand the magnitude of up- and down-regulation, the numbers of unigenes that correspond to infection are also indicated as having 0- to 5-, 5- to 10-, and ≥10- fold changes in expression (Table 1). In slightly regulated groups (0- to 5- fold changes), the number of up-regulated or down-regulated unigenes gradually increased from 6 to 168 hpi. In the regulated groups (5- to 10- fold changes) and greatly regulated groups (≥10- fold changes), the number of up-regulated unigenes increased from 6 to 72 hpi, but decreased thereafter; the number of down-regulated unigenes increased from 6 to 12 hpi, and then decreased at 72 hpi with a final peak at 168 hpi.

**Table 1:** Numbers of up- and down-regulated unigenes at various times post infection compared to CK.

Unigene category	Log2Fold change*	No. of genes			
		6 h	12 h	72 h	168 h
Up-regulated	≥10	14	16	42	39
	5–10	181	139	342	303
	0–5	51	66	101	102
Down-regulated	0–5	52	97	110	128
	5–10	63	364	221	529
	≥10	0	14	8	69
Total regulated		360	696	824	1170

*Probability P value > 0.8.

### Verification of transcriptome data by qPCR

In order to determine the reliability of the transcriptiome sequencing, the relative expression levels of 15 randomly selected genes were analyzed by qPCR (Fig. S3), with the *gapdh* of *S. exigua* used as the reference gene. As can be seen from the comparisons, the qPCR results of these 15 selected genes were almost agreed to the transcriptome data. For example, the CL992.Contig2_ALL was up-regulated in both RNA-seq and qPCR analyses from 6 to 12 hpi, and then down-regulated from 72 to 168 hpi; The CL1118.Contig2_ALL was down-regulated from 6 to 12 hpi, and then up-regulated from 72 to 168 hpi in both the RNA-seq and qPCR analyses; and CL2936.Contig1_ALL was up-regulated in the 6, 12, and 168 hpi samples, and down-regulated at 72 hpi in both RNA-seq and qPCR analyses.

Additional linear regression analysis of the correlation between qPCR and RNA-seq data was performed to investigate the transcriptome data accuracy (Fig. S4). In the 6 h vs CK comparison, the qPCR data showed a significant linear correlation to the RNA-seq data with an R^2^ value of 0.3037, and a Pearson’s r value of 0.5511 (two-tailed P value of 0.0332) (Fig. S4A). In the 12 h vs 6 h comparison, the qPCR data showed a highly significant linear correlation to the RNA-seq data with an R^2^ value of 0.5362, and a Pearson’s r value of 0.7323 (two-tailed P value of 0.0019) (Fig. S4B). In the 72 h vs 12 h comparison, the qPCR data also showed a highly significant linear correlation to the RNA-seq data with an R^2^ value of 0.6475, and a Pearson’s r value of 0.8047 (two-tailed P value of 0.0003) (Fig. S4C). In the 6 h vs CK comparison, the qPCR data showed a highly significant linear correlation to the RNA-seq data with an R^2^ value of 0.7099, and a Pearson’s r value of 0.8426 (two-tailed P value below 0.0001) (Fig. S4D). All of these correlation analyses results suggested a strong positive correlation between the qPCR and transcriptome data.

### DEGs cluster analyses and their possible roles in host response to HvAV-3h

In order to categorize the expression patterns of *S. exigua* unigenes after viral infection, separate cluster analyses of unigenes were performed on the different infectious periods.

#### Cluster analysis in general

Cluster analyses of DEGs produced from different time points vs CK comparisons (6 h vs CK, 12 h vs CK, 72 h vs CK, and 168 h vs CK) were performed to illustrate host larval transcriptional responses during different stages. In total, 87 and 1819 differentially expressed unigenes were found in the intersection and union of different time points vs CK comparisons, respectively (Tables S11,12 The 87 unigenes from intersection cluster analysis can be divided into two major groups: group B1, the down-regulated group; and group B2, the up-regulated group (Fig. 1B). Unigenes in group B1 showed a depressed expression level with a log2Fold change value below −3.12 at all time points. Exceptions were the CL42.Contig6_All, CL3006.Contig1_All, and Unigene18832_All genes (the first three genes in group B1), which were up-regulated at 72 hpi vs CK (Table 2). Sixty-four mutual unigenes (approximately 73.4%) in four different comparisons shared an up-regulation in common (the B2 group), which contained three consistantly highly simulated unigenes (CL3324.Contig3_All, CL3324.Contig1_All, and Unigene12856_All) with a log2Fold change value above 11.60 (Table 2). In the union cluster analysis, two major sub-groups (sub-group C1 and C2) could be divided. Sub-group C1, containing 924 DEGs (approximately 50.8%), were down-regulated in most circumstances, and 13 unigenes exhibited a depressed expression level with no more than −7.37 log2Fold change values in any of the comparisons (Table 2). Furthermore, most of these depressed unigenes could not be annotated with Nr or Swissprot databases, while only three annotated unigenes were calpain-like (CL1931.Contig5_All), lipophorin receptor-like (Unigene3740_All), or cytochrome c oxidase subunit (Unigene25589_All). These annotations may indicate that, the host larval calcium associated signal transduction pathways, which are integral components of membranes, and cytochrome oxidase associated defense mechanisms, were highly inhibited after the HvAV-3h infection. The remaining 49.2% DEGs were grouped into a relatively up-regulated sub-group (C2, Fig. 1C). In this C2 sub-group, twelve highly stimulated unigenes were identified (Table 2), including calcium-sensing receptors (CL3324.Contig3_All, CL3324.Contig1_All), leucine rich repeat protein (Unigene12856_All); fatty acid desaturase 2-like (CL1068.Contig2_All), hypothetical phosphoglycolate phosphatase (CL847.Contig1_All), probable 4a-hydroxytetrahydrobiopterin dehydratase (CL1230.Contig1_All), phosphoribosylformylglycinamidine synthase (CL4264.Contig3_All), and poly [ADP-ribose] polymerase (CL58.Contig3_All). Additionally, the 6 h vs CK and 72 h VS CK DEGs displayed a similar expression pattern and combined as a sub-group (IS2), which was parallel with the 12 h vs CK and 168 h vs CK sub-groups (IS1, Fig. 1B). Whereas, in the union cluster analysis, the 6 h vs CK DEGs were sub-grouped with the 12 h vs CK DEGs (US1), and this sub-group was parallel with the 72 h vs CK and 168 h vs CK DEGs sub-groups (US2, Fig. 1C).

**Table 2:** Extremely expressed unigenes in DEGs cluster analyses of different time point vs CK comparisons.

Unigene	Average FPKM values				Annotation
	6 h vs CK	12 h vs CK	72 h vs CK	168 h vs CK	
Three excption unigenes in sub-group B1, Fig. 3B					
CL42.Contig6_All	−5.03	−3.93	4.4	−4.01	REPAT41 [*Spodoptera exigua*]
CL3006.Contig1_All	−3.42	−5.21	3.51	−5.46	Cuticular proteinprecursor [*Bombyx mori*]
Unigene18832_All	−3.23	−6.59	2.67	−6.63	Cuticular protein precursor [*Bombyx mori*]
Three always highly simulated unigenes in sub-group B2, Fig. 3B					
CL3324.Contig3_All	12.03	11.6	12.51	12.49	KRAB-A domain-containing [*Tinamus guttatus*]
CL3324.Contig1_All	12.75	12.3	13.27	13.16	KRAB-A domain-containing [*Tinamus guttatus*]
Unigene12856_All	13	12.76	13.66	12.23	Uncharacterized protein [*Balaenoptera acutorostrata scammoni*]
Thirteen unigenes with a depressed expression level below 7.37 log2Fold values in sub-group C1, Fig. 3C					
CL3373.Contig2_All	−7.43	−2.09	−7.43	−7.43	NA
Unigene26374_All	−7.78	−1.29	−7.78	−7.78	NA
CL1931.Contig5_All	−8.15	−8.15	−8.15	−8.15	Calpain-B [*Bombyx mori*]
Unigene3740_All	−8.28	−8.28	−8.28	−8.28	Lipophorin receptor protein [*Spodoptera litura*]
Unigene33959_All	−7.51	−7.51	−7.51	−7.51	NA
Unigene27464_All	−7.43	−7.43	−7.43	−7.43	NA
Unigene26275_All	−7.37	−7.37	−7.37	−7.37	NA
Unigene26141_All	−7.4	−7.4	−7.4	−7.4	NA
Unigene26186_All	−7.39	−7.39	−7.39	−7.39	NA
Unigene25589_All	−7.88	−7.88	−7.88	−7.88	Uroplakin-1a [*Tupaia chinensis*]
Unigene21459_All	−7.74	−7.74	−7.74	−7.74	NA
Unigene25368_All	−7.63	−7.63	−7.63	−7.63	NA
Unigene25614_All	−7.63	−7.63	−7.63	−7.63	NA
Twelve highly stimulated unigenes in sub-group C2, Fig. 3C					
CL3324.Contig3_All	12.03	11.6	12.51	12.49	KRAB-A domain-containing protein [*Tinamus guttatus*]
CL3324.Contig1_All	12.75	12.3	13.27	13.16	KRAB-A domain-containing protein [*Tinamus guttatus*]
Unigene12856_All	13	12.76	13.66	12.23	Uncharacterized protein [*Balaenoptera acutorostrata scammoni*]
CL1205.Contig2_All	10.83	10.12	11.24	11.08	NA
CL2935.Contig2_All	10.88	10.4	11.41	11.15	NA
CL2513.Contig5_All	10.97	10.35	11.46	11.36	NA
CL2935.Contig7_All	10.94	10.39	11.38	11.36	NA
CL1068.Contig2_All	10.42	11.47	11.92	11.54	Cytochrome [*Bombyx mori*]
CL847.Contig1_All	10.39	11.44	10.89	10.86	NIPSNAP protein [*Danaus plexippus*]
CL1230.Contig1_All	9.68	9.61	11.8	12.39	Probable pterin-4-alpha-carbinolamine dehydratase [*Bombyx mori*]
CL4264.Contig3_All	9.21	10.24	11.38	11.07	Phosphoribosylformylglycinamidine synthase [*Bombyx mori*]
CL58.Contig3_All	10.13	9.6	10.73	10.64	KRAB-A domain-containing protein [*Tinamus guttatus*]

#### The early infection stage/the host initial response stage (≤12 h post infection)

In the early infection stage, the infected host larvae may exhibit an initial response. In total, 360, 696, and 514 unigenes were involved in the 6 h vs CK, 12 h vs CK, and 12 h vs 6 h cluster analyses, respectively (Fig. S5A–C, Tables S13–15 One-hundred and fourteen (approximately 31.1%, sub-group A1), 475 (approximately 68.4%, sub-group B1), and 404 (approximately 78.6%, sub-group C1) of these DEGs were down-regulated in the 6 h vs CK, 12 h vs CK, and 12 h vs 6 h cluster analyses, respectively. These results may indicate that unigenes were stimulated in the first 6 hpi, and during the 6 to 12 h infectious period, the bulk of DEGs were down-regulated. This was probably due to the host larval initial responses being gradually shifted into initial responses to the ascovirus infection.

**Figure 2:**
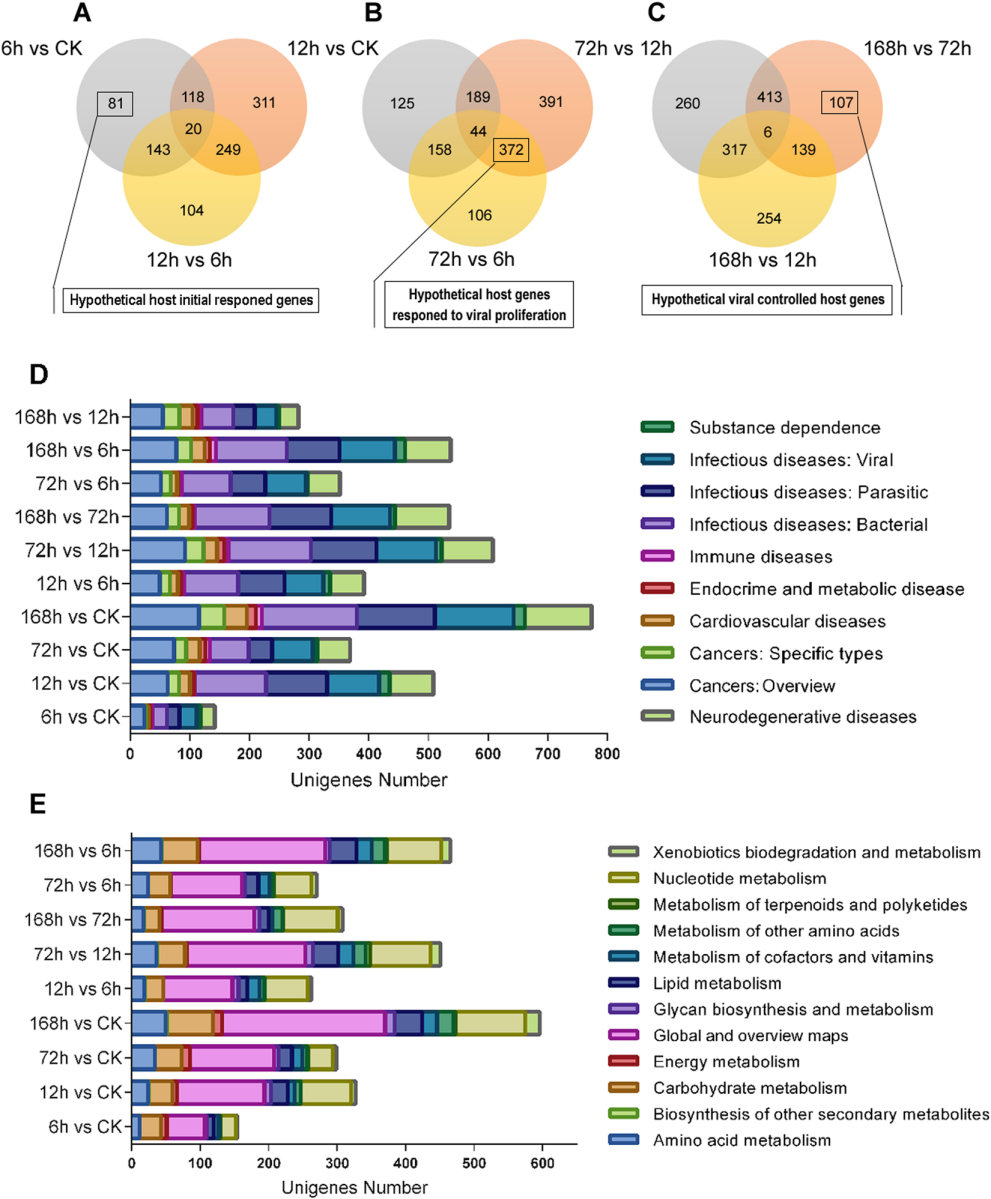
Venn diagrams and KEGG annotation of host larval responding genes in different HvAV-3h infection stages. (**A**) Venn diagram of DEGs from 6 h vs CK, 12 h vs CK and 12 h vs 6 h representing the ascovirus early infectious stage, 81 hypothetical host initial responding genes are illustrated. (**B**) Venn diagram of DEGs from 12 h vs 6 h, 72 h vs 6 h, and 72 h vs 12 h representing the ascovirus mass propagation stage, 372 hypothetical host strong defense responding genes are illustrated. (**C**) Venn diagram of DEGs from 72 h vs 12 h, 168 h vs 12 h, and 168 h vs 72 h representing the ascovirus late infectious stage, 107 hypothetical host late responded genes are illustrated. (**D**) The Diseases in KEGG annotation of host larval DEGs from different comparable groups. (**E**) The Metabolism in KEGG annotation of host larval DEGs from different comparable groups.

Venn diagrams of 6 h vs CK, 12 h vs CK, and 12 h vs 6 h are included to help in understanding the initial response associated DEGs (Fig. 2A). Hypothetical initial response genes were originating from DEGs only found in the 6 h vs CK group (Fig. 2A, gray area, 81 unigenes included). Some of the hypothetical initial response genes were annotated as vesicle-associated membrane protein-like (CL347.Contig1_All), cytochrome P450 6k1-like (Unigene20905_All), UDP-glycosyltransferase (CL1226.Contig1_All), chitinase (CL3948.Contig1_All), ubiquitin-protein ligase (CL3406.Contig8_All), fatty acid synthase-like (CL3472.Contig2_All) (Table S16), which serve to illustrate the general host larval initial responses to ascoviral infection. During the 6 to 12 h infectious period, the host larval initial responses gradually turned into the initial response to HvAV-3h infection. The hypothetical initial response genes were only coming from those genes expressed during the 6 to 12 hpi period. They can be divided into induced initial response genes (Fig. 2A, red area, 311 unigenes included), regulated initial response genes (Fig. 2A, yellow area, 104 unigenes included), and mutual common initial response genes (Fig. 2A, orange area, 249 unigenes included). Further cluster analyses were performed of the intersection of 6 h vs CK and 12 h vs CK (induced initial response genes, 136 unigenes included, Table S17) and 6 h vs CK and 12 h vs 6 h (regulated initial response genes, 163 unigenes included, Table S18) (Fig. S5D,E). The DEGs from the 6 h vs CK and 12 h vs CK groups were clustered into two major sub-groups, the down-regulated sub-group (D1, approximately 41.2%) and the up-regulated sub-group (D2, approximately 59.8%). The DEGs from 6 h vs CK and 12 h vs 6 h, however, were clustered into three major sub-groups: the slightly up-regulated sub-group (E1, approximately 23.2%); the significantly sub-group (E2, approximately 45.1%); and the slightly changed sub-group (E3, approximately 31.7%). These results indicated that during the 6 h–12 h post-infection period, substantial changes in cellular processes, metabolism and other major pathways occurred in host larvae in response to the HvAV-3h infection.

#### The viral mass propagation stage/the host strong defense response stage (12–72 h post infection)

According to the analyses of the unigene expression levels between adjacent infection time points, the 72 h vs 12 h comparison showed a pattern that was reversed from the other times (6 h vs CK, 12 h vs 6 h, and 168 h vs 72 h, Fig. 1A). The 12 h to 72 h interval was a pivotal time period for ascovirus infection. During this time, the hemolymph of the host larvae would turn from a clear liquid (at 12 hpi) into a distinctively milky white color (at 72 hpi), indicating that 60 hours was probably the exponential growth period for ascoviruses. According to our previous research, significant inhibition of host larval growth and feeding cessation began at 24 hpi^13,14^. This would imply that the 12 to 72 h post infection period is a critical stage for both ascoviruses and their host, for it is during this time that both forces seem to be competing for control of the host body. The total of 996 differentially expressed unigenes that were identified during this mid-infection period was clustered into 8 sub-groups according to their expression level (Fig. S6A, Table S19). There were 632 unigenes that were up-regulated (approximately 63.5%, A1-A5 sub-groups) and 364 unigenes were down-regulated (approximately 36.5%, A6-A8 sub-groups). One hundred and fifteen DEGs were annotated with GO terms (Table S20). The most abundant GO terms were: biological regulation (including 34 unigenes), primary metabolic processes (including 34 unigenes), regulation of cellular processes (including 32 unigenes), response to stimuli (including 28 unigenes), and cellular metabolic processes (including 27 unigenes). A total of 655 unigenes were annotated with KEGG terms (Table S21), with the most abundant KEGG pathways being: Metabolic pathways (including 170 unigenes), Amoebiasis (including 95 unigenes), Vibrio cholerea infection (including 87 unigenes), Purine metabolism (including 84 unigenes), and Epstein-Barr virus infection (including 83 unigenes). These results, which support the results of our previous investigation on larval growth inhibition^13^ illustrate that the host larval metabolism during this crucial 12 h to 72 h infection period, may be decisive in the outcome.

Three-hundred and seventy two DEGs were identified in Venn analysis of 12 h vs 6 h, 72 h vs 12 h, and 72 h vs 6 h (orange area of Fig. 2B, Table S22). These unigenes were involved in regulating from 6 h to 72 hpi, but were not involved in the 6 h to 12 hpi period, which may indicate that they were host larval encoded viral proliferation response genes.

#### The very late infection stage/the host larval dying struggle stage (72–168 h post infection)

From 72 hpi on, the host larvae became increasingly moribund (the 72–168 h period, late infection stage). During this stage, larval movement gradually ceased, and their feeding rate decreased precipitiously^13^. Cluster analysis of DEGs from this period included 665 unigenes in total (Table S23), with only 165 up-regulated unigenes (approximately 24.8%, B1-B2 sub-groups), whereas 500 unigenes (approximately 75.2%, B3-B7 sub-groups) were down-regulated (Fig. S6B). There were eight unigenes showing a high up-regulation level with log2Flod values above 10.52; three of these were annotated as binding protein terms (Metabolic pathways, in the Global and overview maps sub-level of the Metabolism level) with KEGG database (Table S24). These results indicated that metabolism was still the cardinal issue that host larvae responded to during the late stage of viral infection. Another Venn diagram analysis showing the 72 h vs 12 h, 168 h vs 72 h and 168 h vs 12 h time periods, shows that 107 hypothetical host larval encoded struggling genes were controlled by the ascovirus during the late infectious stage (red area of Fig. 2C, Table S25).

### GO functional enrichment analysis of DEGs

**Figure 3:**
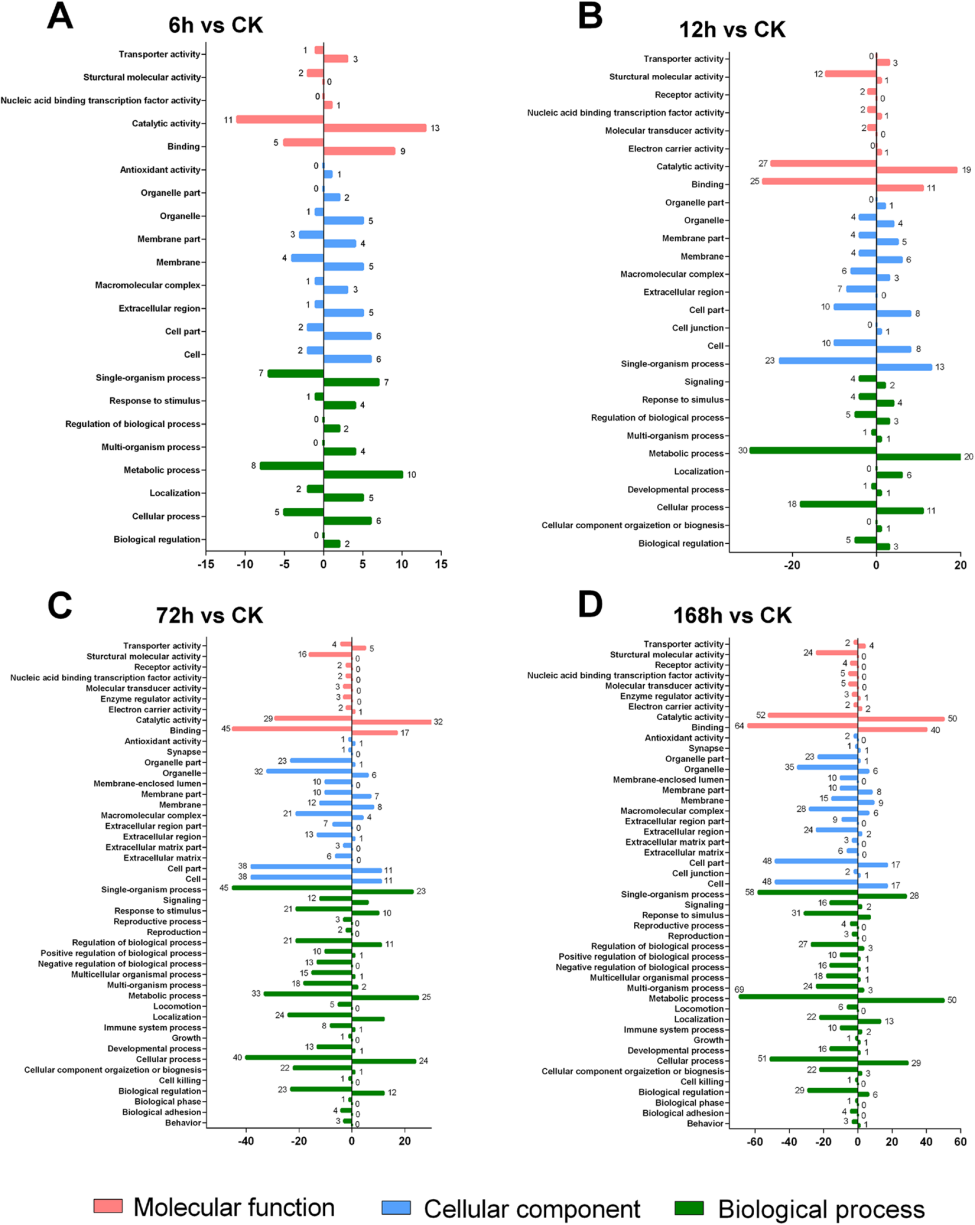
GO enrichment analyses of DEGs among different infected time points compared to CK. GO enrichment analyses of differentially expressed *S. exigua* transcripts were first separated into down-regulated unigenes (left portions of y-axis) and up-regulated unigenes (right portions of y-axis), and then illustrated in 6 h vs CK (**A**), 12 h vs CK (**B**), 72 h vs CK(**C**), and 168 h vs CK (**D**) comparison groups. Red columns represents molecular function, blue columns represents cellular component, and green columns represents biological process.

To understand the functions and biological processes involved in infection, differentially expressed unigenes were enriched to GO terms, as shown in Fig. 3 (Table S26). At 6 hpi, the most abundant GO terms were: metabolic process, single-organism process, cellular process (biological processes); membrane, cell, cell part (cellular components); and catalytic activity, binding, transporter activity (molecular functions) (Fig. 3A, Table S27). At 12 hpi, the most abundant GO terms were: metabolic process, cellular process, biological regulation, regulation of biological process, response to stimulus (biological processes); single-organism process, cell, cell part (cellular components); and catalytic activity, binding, structural molecule activity (molecular functions) (Fig. 3B, Table S28). At 72 hpi, the most abundant GO terms were: single-organism process, cellular process, metabolic process (biological processes); cell, cell part, organelle (cellular components); and binding, catalytic activity, structural molecule activity (molecular functions) (Fig. 3C, Table S29). At 168 hpi, the most abundant GO terms were: metabolic process, single-organism process, cellular process, (biological processes); cell, cell part, organelle (cellular components); and binding, catalytic activity, structural molecule activity (molecular functions) (Fig. 3D, Table S30).

Among the differentially expressed genes in *S. exigua* larvae, the Catalytic activity and Binding were the most abundant up- or down-regulated expressed unigenes enriched GO terms in Molecular process throughout the entail comparisons (Fig. 3). From 12 hpi, the Structural molecular activity annotated unigenes begin to be substantially down-regulated. The Cell, Cell parts, Organelle, and Organelle parts were the most abundant up- or down-regulated expressed unigenes enriched GO terms in Cellular components from 12 to 168 hpi. Single-organism processes, Metabolic processes, and Cellular processes.

Cellular components and biological processes were the most abundant up- or down-regulated expressed unigenes enriched GO terms in Molecular processes throughout all the comparisons.

### Functional enrichment analysis by KEGG pathways

**Figure 4:**
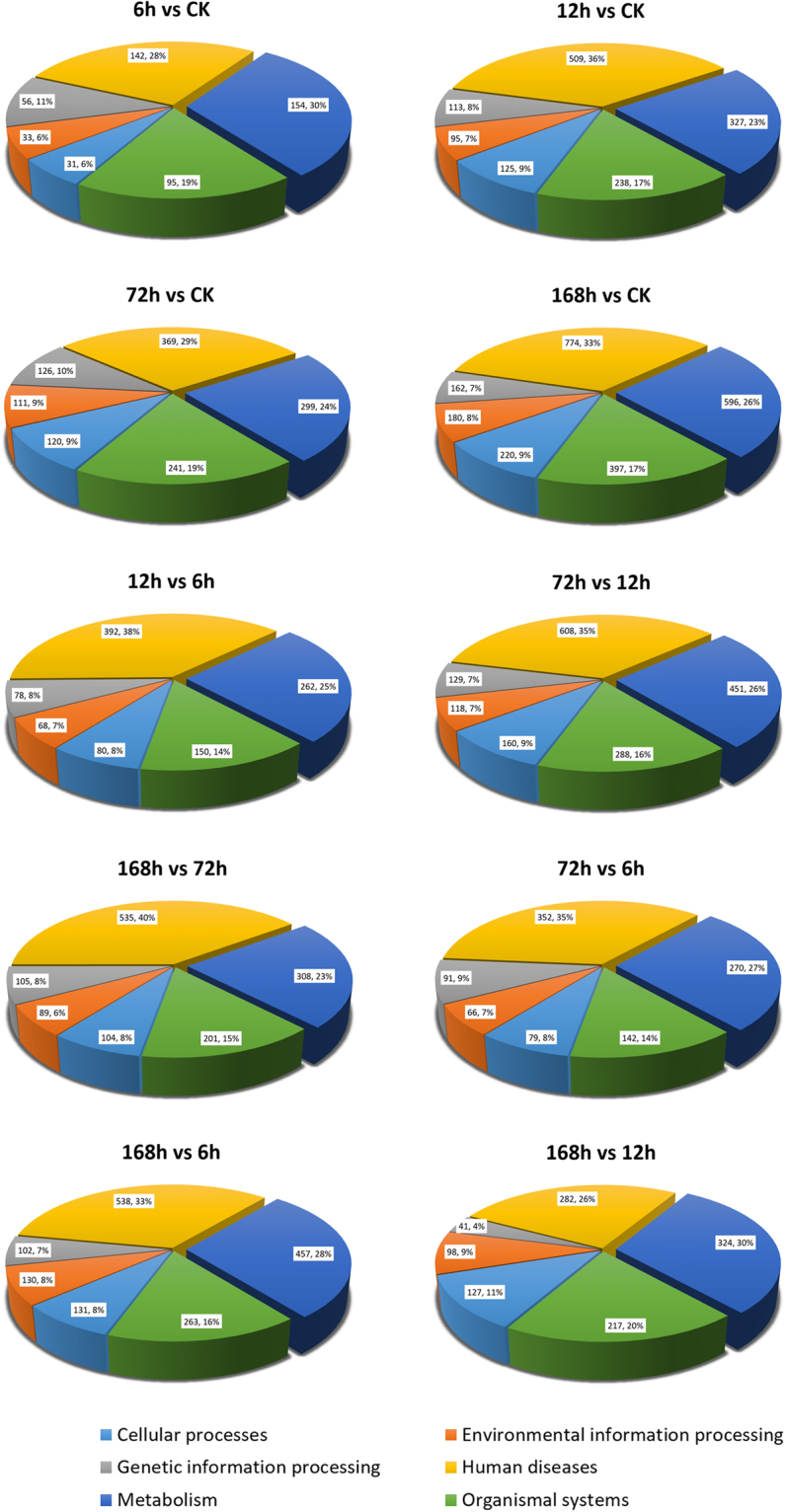
KEGG analysis of *S. exigua* differentially expressed unigenes from different comparable groups. The DEG numbers and the percentage of each sub-level are labeled on each of the parts.

To better understand the host larval responded unigenes’ functions, the DEGs between different groups were enriched to KEGG terms (Fig. 4, Tables S31–35 As can be seen from Fig. 4, genes enriched into metabolism, organismal systems, and human diseases occupy the top three proportions; while DEGs enriched into genetic cellular processes, information processing, and environmental information processing terms combined, occupy no more than one third of the total area in any of the pie chart comparisons.

#### Pathways involving cellular processes

For the cellular processes (the light blue portions of the pie charts, Fig. 4) pathway level, unigenes were mainly enriched into four sub-levels, including cell growth and death, cell motility, cellular community, and transport and catabolism. Oocyte meiosis, Apoptosis, P53 signaling pathway, and Cell cycle were included in the Cell growth and death sub-level, while regulation of Actin cytoskeleton was identified in the Cell motility sub-level (Table 3). Obviously, more DEGs were annotated to regulation of actin cytoskeleton from 12 hpi, compared to 6 hpi. All of the differentially expressed unigenes annotated into the cell growth and cell motility sub-levels of the cellular processes pathway are detailed in Table 3.

**Table 3:** Most abundant DEGs enriched cellular processes pathways in KEGG.

Comparison	Pathway	DEG Number	P value	Pathway ID	Sub-level	Unigenes
6 h vs CK	Oocyte meiosis	3	0.29	ko04114	Cell growth and death	CL3324.Contig1_All, CL3324.Contig3_All, Unigene19404_All
	Apoptosis	2	0.47	ko04210	Cell growth and death	CL375.Contig6_All, CL6434.Contig13_All
	p53 signaling pathway	1	0.66	ko04115	Cell growth and death	CL2926.Contig4_All
	Cell cycle	1	0.86	ko04110	Cell growth and death	Unigene18832_All
	Regulation of actin cytoskeleton	4	0.97	ko04810	Cell motility	Unigene14456_All, Unigene6183_All, CL6081.Contig3_All, CL1053.Contig1_All
12 h vs CK	p53 signaling pathway	4	0.27	ko04115	Cell growth and death	CL46.Contig1_All, CL46.Contig4_All, CL46.Contig3_All, CL46.Contig12_All
	Oocyte meiosis	5	0.50	ko04114	Cell growth and death	CL3324.Contig1_All, CL3324.Contig3_All, Unigene19404_All, Unigene10341_All, CL2851.Contig3_All
	Apoptosis	3	0.74	ko04210	Cell growth and death	Unigene4757_All, CL46.Contig6_All, Unigene8172_All
	Cell cycle	3	0.86	ko04110	Cell growth and death	Unigene18832_All, Unigene24283_All, Unigene10341_All
	Regulation of actin cytoskeleton	23	0.35	ko04810	Cell motility	CL2386.Contig2_All, Unigene15718_All, Unigene7475_All, Unigene6183_All, CL6091.Contig28_All, CL774.Contig3_All, Unigene10070_All, CL4153.Contig2_All, CL2386.Contig1_All, CL4277.Contig4_All, CL6091.Contig17_All, Unigene881_All, Unigene21742_All, Unigene7424_All, Unigene4361_All, Unigene16131_All, Unigene15581_All, Unigene13774_All, CL1853.Contig1_All, Unigene10341_All, Unigene17975_All, Unigene2176_All, Unigene15493_All
72 h vs CK	Regulation of actin cytoskeleton	27	0.26	ko04810	Cell motility	CL4779.Contig1_All, CL304.Contig1_All, CL1053.Contig3_All, Unigene15718_All, Unigene6183_All, CL6091.Contig28_All, Unigene11423_All, Unigene837_All, CL6081.Contig3_All, CL1007.Contig9_All, CL774.Contig3_All, CL6163.Contig1_All, CL1937.Contig1_All, Unigene15208_All, Unigene21742_All, CL673.Contig2_All, Unigene5697_All, Unigene15581_All, Unigene836_All, CL1053.Contig1_All, Unigene1017_All, CL2101.Contig2_All, CL5337.Contig2_All, Unigene11370_All, Unigene2176_All, CL1823.Contig3_All, CL6091.Contig9_All
	Cell cycle	5	0.63	ko04110	Cell growth and death	CL639.Contig3_All, CL2725.Contig1_All, Unigene4970_All, Unigene18832_All, Unigene24283_All
	Oocyte meiosis	4	0.77	ko04114	Cell growth and death	CL3324.Contig1_All, CL1937.Contig1_All, CL3324.Contig3_All, Unigene19404_All
	p53 signaling pathway	2	0.80	ko04115	Cell growth and death	Unigene19495_All, CL2926.Contig4_All
	Apoptosis	2	0.93	ko04210	Cell growth and death	CL6434.Contig8_All, CL512.Contig6_All
168 h vs CK	p53 signaling pathway	8	0.085	ko04115	Cell growth and death	CL292.Contig9_All, CL46.Contig1_All, CL46.Contig4_All, CL46.Contig3_All, CL46.Contig12_All, Unigene19495_All, CL2926.Contig4_All, CL3077.Contig3_All
	Oocyte meiosis	5	0.90	ko04114	Cell growth and death	CL3324.Contig1_All, CL3324.Contig3_All, Unigene19404_All, Unigene10341_All, CL2851.Contig3_All
	Cell cycle	5	0.91	ko04110	Cell growth and death	CL639.Contig3_All, CL2725.Contig1_All, Unigene18832_All, Unigene24283_All, Unigene10341_All
	Apoptosis	3	0.96	ko04210	Cell growth and death	Unigene4757_All, CL46.Contig6_All, Unigene8172_All
	Regulation of actin cytoskeleton	50	0.01	ko04810	Cell motility	CL4779.Contig1_All, CL3655.Contig1_All, CL2386.Contig2_All, Unigene17949_All, Unigene15718_All, CL1569.Contig5_All, Unigene7475_All, Unigene6183_All, CL6091.Contig28_All, Unigene11423_All, CL3212.Contig2_All, Unigene25270_All, Unigene837_All, CL3212.Contig1_All, CL1063.Contig1_All, CL774.Contig3_All, CL6163.Contig1_All, Unigene10070_All, Unigene2661_All, CL4153.Contig2_All, CL2386.Contig1_All, Unigene11289_All, CL1187.Contig2_All, Unigene881_All, Unigene872_All, Unigene19351_All, Unigene15208_All, Unigene21742_All, Unigene7424_All, Unigene4361_All, Unigene17223_All, CL5420.Contig3_All, Unigene16131_All, CL6091.Contig2_All, Unigene15581_All, CL5420.Contig2_All, Unigene836_All, Unigene1902_All, CL3685.Contig1_All, Unigene5296_All, Unigene5872_All, Unigene13774_All, Unigene11370_All, Unigene17826_All, CL1853.Contig1_All, Unigene10341_All, CL3502.Contig2_All, CL4812.Contig3_All, CL3212.Contig3_All, CL6091.Contig9_All

#### Pathways involving Organismal systems

DEGs enriched into Organismal systems (the green portions in the pie charts, Fig. 4) occupy from 14% to 20% of the area in the KEGG enrichment comparisons. This classification was further subdivided into Circulatory system, Development, Digestive system, Endocrine system, Environmental adaptation, Excretory system, Immune system, Nervous system, and Sensory system. As can be seen from Fig. S7, most unigenes were annotated into the Digestive system, Endocrine system, Immune system, Development, and Nervous system. Compared to the CK, the DEGs from different time points showed a similar enrichment, with the DEG numbers increasing relative to the time duration (Fig. S7A). In the analysis of the Organismal system enrichment between adjacent infection time points, the greatest enrichment occurred in the 72 h vs 12 comparisons (the gray line in Fig. S7B), in which the endocrine system (77, 11.7% in KEGG annotated DEGs of 72 h vs 12 h comparison), immune system (66, 10.1% in KEGG annotated DEGs of 72 h vs 12 h comparison), and digestive system (58, 8.9% in KEGG annotated DEGs of 72 h vs 12 h comparison) comprised a large percentage of the total in this period analysis. The specific unigenes involved in the 12 h to 72 h infection period enriched into organismal system are shown in Table S36. Obviously, Axon guidance, Osteoclast differentiation, and Dorso-ventral axis formation were the most modified pathways involving unigenes in the Development sublevel; Protein digestion and absorption, Pancreatic secretion, and Salivary secretion were the most modified unigenes involved pathways in the Digestive system sublevel; Thyroid hormone signaling pathway, PPAR signaling pathway, and Insulin signaling pathway were the most modified unigenes involved pathways in the Endocrine system; and Platelet activation, Leukocyte transendothelial migration, and Chemokine signaling pathway were the most modified unigenes involved pathways in the immune system sublevel (Table S36). DEGs from other time point comparisons are illustrated in Fig. S7C. Similar to the enrichment analysis above, the most abundant pathways were annotated into the Development, Digestive system, Endocrine system, Immune system, and Nervous system. Each of these results indicates that, the host larval Development, Digestive system, Endocrine system, Immune system, and Nervous system were the prime viral attack targets.

#### Metabolism pathways

The Metabolism terms (the dark blue portions in the pie charts, Fig. 4) were another large component in the KEGG enrichment analysis. In this term, the most abundant sub-level pathways were: Global and overview maps, Nucleotide metabolism, and Carbohydrate metabolism (Fig. 2E, Tables S37–40 To help in understanding the host larval metabolic changes following HvAV-3h infection, the three most abundant metabolism pathways in each of the sub-level terms are illustrated in Tables S41–44 yrosine metabolism may be a key pathway involved in Amino acid metabolism throughout all of the infectious stages because it was consistantly found in the top three most abundant Amino acid metabolisms in the 6 h, 12 h, 72 h, and 168 h vs CK DEGs; Cysteine and methionine metabolism may also be a key pathway in Amino acid metabolism from within the 72 h infectious stage; and Glycine, serine and threonine metabolism was probably a key pathway involved in Amino acid metabolism in the 12 h to 168 h infectious stage. Amino sugar and nucleotide sugar metabolism and Pyruvate metabolism were the most abundant terms in Carbohydrate metabolism, indicating that these two pathways were mainly infected during the entirety of the HvAV-3h infection stages. Several lipid metabolism pathways such as Fatty acid metabolism, Fatty acid degradation, and Glycolipid metabolism were found to be the three most abundant pathways in Global and overview maps and Lipid metabolism sub-levels, which indicated that the formation of HvAV-3h vesicles may be associated with these pathways. Insect hormone biosynthesis in the metabolism of terpenoids and polyketides sub-level were only found in the 72 h vs CK and 168 h vs CK analyses, which indicated that insect hormones such as juvenile hormone or ecdysone were influenced in the late ascovirus infectious stages. In summary, all of the DEGs enriched into metabolism pathways provide a comprehensive view of the physiological disruption that occurs within the host larvae following infection by HvAV-3h.

## Discussion

HvAV-3h infection of susceptible noctuid larvae such as *S. litura, S. exigua*, and *H. armigera* leads to a significant reduction in larval feeding and inhibition of larval growth and weight gain^13,14^. Typical vesicals containing numerous virions were observed in ascovirus infected larval tissues (fat bodies or hemolymph)^7,12^. Most previous research on ascoviruses have focused on aspects of their unique life-cycle, including their encoded caspase-like or inhibitor of apoptosis protein-like functional^5–7,25–27^. Not nearly as well known as other large dsDNA insect viruses (such as baculoviruses), few other studies involving detailed interactions between ascoviruses and their host larvae have been reported. A transcriptome data analysis of *Spodoptera frugiperda ascovirus* (SfAV-1a) was recently published, in which the SfAV-1a encoded genes were analyzed in detail providing a special insight into the molecular biology of an ascovirus^28^. In the present study, the host larval transcriptome response was determined, in which a comprehensive overview of transcription level regulation corresponding to the HvAV-3h infection was discussed.

Ascoviruses were reported as insect-pathogens that can attack the larval or pupal stages of lepidopterous insects, causing an prolonged but ultimately fatal disease^12–14,27^. When infected by HvAV-3h, *S. exigua* and *H. armigera* larvae will normally spend an extra 5 to 20 days in a morbid or catatonic state before succumbing to the virus^12^. Similarly extended larval stages have been noted in baculovirus infected lepidopteran caterpillars. The reasons stated for causing the larva’s inactive condition were due to the ecdysteroid UDP-Glucosyl transferase (EGT) protein encoded by the baculovirus leading to inactivation of the host larval ecdysteroids^15,20,29,30^. Although there was no hypothetical EGT homologue encoded by ascoviruses, there are still similarities in the pathogenic courses between ascoviruses and baculoviruses. Recent transcriptome research on SfAV-1a indicated ascoviruses possess a transcription pattern similar to other large DNA viruses such as baculoviruses. They divided the SfAV-2a gene transcription into three groups: early (genes expressed from 6 hpi), late (genes expressed from 12 hpi) and very late genes (genes expressed from 24 hpi)^20,28^. In this study, we divided the ascovirus infection and host larval response stage into three groups as well, but with differing time durations: the host initial response stage (early infection stage, up to the first 12 hpi); the host strong defense response stage (viral mass propagation stage, from 12–72 hpi), and the host larval dying struggle stage (very late infection stage, from 72–168 hpi).

In the early infection stage, 31.1% of DEGs were down-regulated in the initial 6 hpi, with the down-regulated DEGs portion increasing to approximately 68.4% during the 6–12 hpi period (Fig. S5A,B). A similar regulation tendency was found in *Autographa californica* multiple nucleopolyhedrovirus (AcMNPV) infected *Trichoplusia ni* cell lines, where more down-regulated unigenes were found at 12 hpi compared to 6 hpi^31^. We also identified 81 hypothetical initial response genes, such as vesicle-associated membrane protein-like encoding gene (CL347.Contig1_All), cytochrome P450 encoding gene (Unigene20905_All), UDP-glycosyltransferase encoding gene (CL1226.Contig1_All), chitinase encoding gene (CL3948.Contig1_All), ubiquitin-protein ligase encoding gene (CL3406.Contig8_All), and fatty acid synthase-like encoding gene (CL3472.Contig2_All) (Table S16). Interestingly, no heat shock protein genes (*hsp*) were annotated in the hypothetical initial response genes. *Hsp70* was found to be slightly induced in AcMNPV infected Sf9 cells and possibly important for viral DNA replication^31–33^, while *hsp70* was greatly down-regulated in AcMNPV infected *T. ni* cells. These results indicate that ascoviruses may follow DNA replication rules that differ from those of baculoviruses, and that HSP is not involved in *S. exigua* larval initial responses to HvAV-3h infection. Several typical insect immune pathways were identified in the host initial response stage, such as Toll-like receptor signaling pathway and Jak-STAT signaling pathway, both of which have been well documented in other insects including *Drosophila melanogaster Bombyx mori, Manduca sexta, Aedes aegypti, Hepialus xiaojinenxix*, and *Tribolium castaneum*^34–39^. Compared to human diseases, abundant host regulated unigenes were annotated during the first 12 hpi as Immune diseases, Infectious diseases: Bacterial, Infectious diseases: Parasitic, Infectious diseases: Viral, and Neurodegenerative diseases, indicating that the ascoviral pathogenicity was somewhat similar to human diseases. As recent studies have reported, the insect host innate immune responses may also play a pivotal role in viral infections^40–43^, as in the *hsp70* mentioned above, ascovirus infection may be associated with different host larval innate immune responses.

As viruses are dependent on their insect host to provide energy and resources for viral replication and assembly, the host larval metabolism metabolic pathways would be tremendously regulated during the infection processes^44,45^. In our study, KEGG analysis demonstrated that the Metabolic were the largely DEGs enriched pathways (Fig. 4). Except in the Global and overview maps, Nucleotide metabolism, Carbohydrate metabolism, Amino acid metabolism, and Lipid metabolism were the most abundant sublevels in Metabolism enrichment analysis (Fig. 2E). All biological processes, such as development, differentiation, or proliferation, etc., require the substrates or energy derived from metabolism. It has been reported that the metabolic adaptations for uptake, storage, and utilization of substrates are largely controlled by extracellular signals such as insulin^46^. Unigenes associated with Insulin secretion and Insulin signaling pathway were identified from the 6 h to 168 h versus CK KEGG enrichment analysis, and insulin resistance associated unigenes were found in the 12 h to 168 h versus CK KEGG enrichment. These results suggest that the insulin associated pathways may be a possible means for HvAV-3h to disrupt the host larval metabolism. Additionally, it is believed that metabolism, especially lipid metabolism, is closely related to host immune responses^47^, including the host antiviral innate responses^48,49^. Because the immune cells lack significant stores of nutrients, these effector responses can only be sustained if immune cells can dramatically increase the uptake of glucose, amino acids, and fatty acids from their microenvironment^50–52^. In our study, numerous differentially expressed unigenes were found in lipid metabolism pathways, including Biosynthesis of unsaturated fatty acids, Fatty acid metabolism, Fatty acid degradation, Fatty acid elongation, Fat digestion and absorption, Glycerolipid metabolism, Ether lipid metabolism, Sphingolipid metabolism, Glycerophospholipid metabolism, etc. In addition to their presumably important roles in the host immune responses to HvAV-3h infection, the lipid metabolism might also be associated with the formation of ascoviral vesicles.

As we had previously mentioned, the Toll-like receptors (TLRs) were the first to be identified and have been the most thoroughly studied of the host initial immune response pathways. Together with RIG-I-like receptors (RLRs), NOD-like receptors, C-type lectin receptors and sequestosome 1/p62-like receptors, they are all host encoded classical PRRs (pattern recognition receptors), which can be distinguished from host components within hours after being invaded by pathogens^53^. Those PPRs associated DNA sensor pathways would activate downstream transcription factors, leading to the induction of type I interferon (IFN), inflammatory cytokines and chemokines^54,55^. In our study, a total of 83, 23, and 15 unigenes were annotated as interferon, inflammatory cytokines and chemokines associated genes (Table S3). The induction of these antiviral responses is dependent upon coordinated activation of the latent cytosolic transcriptional factors such as the IFN regulatory factors (IRFs, including IRF3 and IRF 7), and nuclear factor-kappa B (NF-κB)^56^. IFNs act in a paracrine autocrine fashion to activate the JAK-STAT pathways, up-regulating the transcription of hundreds of IFN-stimulated genes (ISGs), many of which possess broad antiviral activities^53,57^. The NF-κB signaling pathway was found throughout the DEGs KEGG annotation, while the NOD-like receptor signaling pathway was found only in the 6 h vs CK DEGs KEGG enrichment. The JAK-STAT signaling pathway was found in all except in the 6 h vs CK DEGs KEGG enrichment (Table 4). In summary, based on our transcriptome data, the *S. exigua* larvae produced an intense defensive response toward HvAV-3h, as shown by their metabolic regulation together with their immune responses being strongly adapted in different associated classical pathways.

**Table 4:** Immunity pathways in different comparisons.

Comparisons	Pathway	DEG Number	P Value	Pathway ID	Sub-level 1	Sub-level 2	Unigenes
6 h vs CK	Insulin secretion	5	0.02	ko04911	Organismal Systems	Endocrine system	CL3324.Contig1_All, CL3324.Contig3_All, Unigene705_All, Unigene19404_All, CL347.Contig1_All
	Insulin signaling pathway	2	0.91	ko04910	Organismal Systems	Endocrine system	CL1681.Contig2_All, CL3472.Contig2_All
	NF-kappa B signaling pathway	5	0.013	ko04064	Environmental Information Processing	Signal transduction	CL375.Contig6_All, CL3324.Contig1_All, Unigene8993_All, CL3324.Contig3_All, CL58.Contig3_All
	NOD-like receptor signaling pathway	1	0.63	ko04621	Organismal Systems	Immune system	CL375.Contig6_All
12 h vs CK	Insulin secretion	5	0.33	ko04911	Organismal Systems	Endocrine system	CL3324.Contig1_All, CL3324.Contig3_All, Unigene705_All, Unigene19404_All, Unigene19354_All
	Insulin signaling pathway	8	0.76	ko04910	Organismal Systems	Endocrine system	CL1681.Contig2_All, Unigene4757_All, Unigene10070_All, CL46.Contig6_All, Unigene8172_All, CL1853.Contig1_All, Unigene10341_All, CL2851.Contig3_All
	Insulin resistance	4	0.87	ko04931	Human Diseases	Endocrine and metabolic diseases	Unigene4757_All, CL46.Contig6_All, Unigene8172_All, CL1853.Contig1_All
	NF-kappa B signaling pathway	5	0.25	ko04064	Environmental Information Processing	Signal transduction	CL3324.Contig1_All, Unigene8993_All, CL3324.Contig3_All, Unigene11306_All, CL58.Contig3_All
	Jak-STAT signaling pathway	6	0.10	ko04630	Environmental Information Processing	Signal transduction	Unigene4757_All, Unigene10070_All, CL46.Contig6_All, Unigene8172_All, Unigene24283_All, Unigene10341_All
72 h vs CK	Insulin secretion	5	0.43	ko04911	Organismal Systems	Endocrine system	CL3324.Contig1_All, CL3324.Contig3_All, Unigene705_All, Unigene19404_All, Unigene11630_All
	Insulin resistance	2	0.99	ko04931	Human Diseases	Endocrine and metabolic diseases	CL1681.Contig2_All, CL463.Contig1_All, CL1937.Contig1_All
	Insulin signaling pathway	3	1.00	ko04910	Organismal Systems	Endocrine system	CL1681.Contig2_All, CL463.Contig1_All, CL1937.Contig1_All
	NF-kappa B signaling pathway	6	0.18	ko04064	Environmental Information Processing	Signal transduction	CL6163.Contig1_All, CL3324.Contig1_All, Unigene8993_All, CL3324.Contig3_All, Unigene11306_All, CL58.Contig3_All
	Jak-STAT signaling pathway	3	0.69	ko04630	Environmental Information Processing		CL639.Contig3_All, Unigene4970_All, Unigene24283_All
168 h vs CK	Insulin secretion	6	0.63	ko04911	Organismal Systems	Endocrine system	CL3324.Contig1_All, Unigene19278_All, CL3324.Contig3_All, Unigene705_All, Unigene19404_All, Unigene19354_All
	Insulin resistance	6	0.95	ko04931	Human Diseases	Endocrine and metabolic diseases	Unigene4757_All, CL1842.Contig6_All, CL46.Contig6_All, Unigene8172_All, CL1197.Contig3_All, CL1853.Contig1_All
	Insulin signaling pathway	10	0.97	ko04910	Organismal Systems	Endocrine system	CL1681.Contig2_All, CL292.Contig9_All, Unigene4757_All, Unigene15555_All, Unigene10070_All, CL46.Contig6_All, Unigene8172_All, CL1853.Contig1_All, Unigene10341_All, CL2851.Contig3_All
	NF-kappa B signaling pathway	6	0.51	ko04064	Environmental Information Processing	Signal transduction	CL6163.Contig1_All, CL3324.Contig1_All, Unigene8993_All, CL3324.Contig3_All, CL3534.Contig1_All, CL58.Contig3_All
	Jak-STAT signaling pathway	7	0.29	ko04630	Environmental Information Processing	Signal transduction	Unigene4757_All, CL639.Contig3_All, Unigene10070_All, CL46.Contig6_All, Unigene8172_All, Unigene24283_All, Unigene10341_All

In conclusion, the interactions between the host and ascovirus were comprehensively delineated via transcriptome analysis in our study. From pathways to specific unigenes, and from the initial to late infection stages, we discussed the processes involved in host responses to ascovirusal infections. It is hoped that these results will benefit future studies concerning ascoviral infection processes, and the pathogenic properties of ascoviruses.

## Materials and Methods

### Viruses and larvae

Heliothis virescens ascovirus 3 h (HvAV-3h) was isolated by Huang *et al*. and stored in Huang’ s laboratory^7^. A laboratory colony of beet armyworm, *Spodoptera exigua*, was cultured on an artificial diet at 27 ± 1 °C and a 16/8 h (light/dark) photoperiod.

### Infection of the *S. exigua* larvae with HvAV-3h

The titer of HvAV-3h containing hemolymph was determined by end-point dilution method^7,14^. The HvAV-3h virion at a multiplicity of infection (M.O.I.) of 150–200 virions/cell was used to inoculate the *S. exigua* larvae. A sterile insect pin dipped into the HvAV-3h hemolymph was used to pierce the proleg of newly molted third instar larvae. The inoculated larvae were then reared separately on artificial diet dots until the preset time for sampling. Control larvae were handled identically except the pins used for injection were dipped into PBS instead of HvAV-3h hemolymph.

### Total RNA extraction

In order to obtain the most consistant transcription information, whole body homogenates of *S. exigua* larvae at 6, 12, 72, and 168 hpi were used in RNA extraction for the T1 (6 hpi), T2 (12 hpi), T3 (72 hpi), and T4 (168 hpi) groups, respectively. The control larvae, which were injected with PBS for 24 hours as described above, were used for RNA extraction of the CK group. Total RNA was extracted from each sample using TRI REAGENT (Molecular Research Center, INC, Ohio, USA) following the manufacturer’s instructions. The RNA extracted from each sample was quantified by measuring the OD values at A260/A280 using a Nano Drop 2000 spectrophotometer and agarose gel electrophoresis. Three biological replicates were conducted for all group samples (CK, T1, T2, T3, and T4) and used for independent library preparations.

### Transcription mRNA sequencing, assembling, and unigene annotation

Library constructions and RNA sequencing were performed by the Beijing Genomics Institute (Shenzhen, CHN). Briefly, the DNA in the total RNA was removed by DNase I digestion, and the mRNA was purified by Oligo (dT) conjunct breads; the enriched mRNAs were broken into small pieces using a Thermomixer, and the broken mRNAs used to synthetize cDNAs followed by cDNA purification, cohesive end reparation; cDNA library quantification was performed using an Agilent 2011 Bioanalyzer and the ABI StepOnePlus Real-Time PCR System. Finally, the cDNA libraries were sequenced on an Illumaina HiSeq^TM^ 4000.

The clean reads were filtered from the the raw reads by removing the reads with low quality, reads containing adapters, or reads with unknown nucleotides >5%. The clean reads were de novo assembled by Trinity (version v2.0.6), followed by clustering to redundancy with Tgicl (version v2.0.6) to obtain unigenes. Blast (http://blast.ncbi.nlm.nih.gov/Blast.cgi) was used to annotate NT, NR (ftp://ftp.ncbi.nlm.nih.gov/blast/db), COG (http://www.ncbi.nlm.nih.gov/COG), KEGG (http://www.genome.jp/kegg), and SwissProt (http://ftp.ebi.ac.uk/pub/databases/swissprot); Blast2GO (https://www.blast2go.com) was used to annotate NR and GO (http://geneontology.org)^58^; InterProScan5 (https://code.google.com/p/interproscan/wiki/Introduction) were used to annotate InterPro (http://www.ebi.ac.uk/interpro)^59^.

### Identification of differentially expressed genes (DEGs) and cluster analysis

To identify differentially expressed unigenes, the cleaned reads were aligned to the assembled unigenes using Bowtie2 (v2.2.5, http://bowtie-bio.sourceforge.net/Bowtie2/index.shtml)^60^, and the unigenes’ expression levels were calculated by RSEM (v1.2.12, http://deweylab.biostat.wisc.edu/RSEM)^61^. NOIseq method was used to detect the differentially expressed genes with a Log2Fold change value ≥2.00 and Probability ≥0.8. The cluster analyses of DEGs were performed with pheatmap function in the R software, the intersection DEGs and union DEGs between different comparisons were both analyzed separately. The following enrichment analyses of DEGs were performed according to GO and KEGG annotation results with phyper function in the R software, the P values were calculated as described at https://en.wikipedia.org/wiki/Hypergeometric_distribution.

### Validation of RNA-Seq data by quantitative RT-PCR

Fifteen genes were selected for confirmation by real-time PCR (RT-qPCR) using 2 × SYBR green real-time PCR mix (TaKaRa) according to the manufacturer’s protocol, and *gapdh* was used as the reference gene (NCBI accession NO. JF728815.1 or transcriptome unigene Unigene15875_All). The prepared total RNA used in RT-PCR analysis was isolated from three biological replicate samples as that used for RNA-seq. The qPCR was performed on the Bio-Red CFX9600 real-time PCR system (Bio Red, USA). The primers (Table S45) used for qPCR of selected genes were designed according to RNA-seq data with Primer Premier 5 (PREMIER Biosoft international, Palo Alto, CA, USA). The PCR amplification was performed in triplicate, using the following cycling parameters: 94 °C for 2 min, followed by 40 cycles of 15 s at 94 °C, and then 20 s at 60 °C. All samples were analyzed in triplicate and the relative target gene expression levels were calculated by the 2^−ΔΔCT^ method and then the Log2Fold values between different comparisons were calculated and compared with the transcriptome data. Additional correlation analyses between the transcriptome data and qPCR data were performed by using two-tailed Pearson methods in SPSS 15.0.

### Data Availability

The datasets generated during and/or analysed during the current study are available from the corresponding author on reasonable request.

## Electronic supplementary material

Supplemental file 1

Supplemental file 2

Supplemental file 3

## References

[1] Cheng X, Carner GR, Brown TM (1999). Circular configuration of the genome of ascoviruses. J Gen Virol..

[2] Federici BA, Govindarajan R (1990). Comparative histopathology of three Ascovirus isolates in larval noctuids. J Invertebr Pathol..

[3] Federici BA (1983). Enveloped double-stranded DNA insect virus with novel structure and cytopathology. Proc Natl Acad Sci USA.

[4] Huang GH, Hou DH, Wang M, Cheng XW, Hu Z (2017). Genome analysis of Heliothis virescens ascovirus 3h isolated from China. Virologica Sinica.

[5] Asgari S (2007). A caspase-like gene from Heliothis virescens ascovirus (HvAV-3e) is not involved in apoptosis but is essential for virus replication. Virus Res..

[6] Wei YL (2014). 2014. Genome sequence and organization analysis of Heliothis virecens ascovirus 3f isolated form a *Helicoverpa zea* larva. J Invertebr Pathol..

[7] Huang GH (2012). Phylogenetic position and replication kinetics of Heliothis virescens ascovirus 3h (HvAV-3h) isolated from *Spodoptera exigua*. PLoS ONE.

[8] Furlong MJ, Asgari S (2010). Effects of an ascovirus (HvAV-3e) on diamondback moth, *Plutella xylostella* and evidence for virus transmission by a larval parasitoid. J Invertebr Pathol..

[9] Li SJ (2016). Imperfection works: survival, transmission and persistence in the system of Heliothis virescens ascovirus 3h (HvAV-3h), *Microplitis similis* and *Spodoptera exigua*. Sci Rep..

[10] Stasiak K, Renault S, Federici BA, Bigot Y (2005). Characteristics of pathogenic and mutualistic relationships of ascoviruses in field populations of parasitoid wasps. J Insect Physiol..

[11] Asgari S (2006). Replication of Heliothis virescens ascovirus in insect cell lines. Arch Virol..

[12] Govindarajan R, Federici BA (1990). Ascovirus infectivity and effects of infection on the growth and development of noctuid larvae. J Invertebr Pathol..

[13] Hu J (2016). Characterization and Growing Development of *Spodoptera exigua* (Lepidoptera: Noctuidae) Larvae Infected by Heliothis virescens ascovirus 3h (HvAV-3h). J Econ Entomol..

[14] Li SJ (2013). A comparison of growth and development of three major agricultural insect pests infected with Heliothis virescens ascovirus 3 h (HvAV-3h). PLoS ONE.

[15] O’Reilly DR, Miller LK (1989). A baculovirus blocks insect molting by producing ecdysteroid UDP-glucosyl transferase. Science.

[16] Shikata M (1998). The ecdysteroid UDP-glucosyltransferase gene of *Autographa californica* nucleopolyhedrovirus alters the moulting and metamorphosis of a non-target insect, the silkworm, *Bombyx mori* (Lepidoptera, Bombycidae). J Gen Virol..

[17] Iwanaga M, Shimada T, Kobayashi M, Kang WK (2007). Identification of differentially expressed host genes in *Bombyx mori* nucleopolyhedrovirus infected cells by using subtractive hybridization. Appl Entomol Zool..

[18] Bao YY (2010). Comparative analysis of *Bombyx mori* nucleopolyhedrovirus responsive genes in fat body and haemocyte of *B. mori* resistant and susceptible strains. Insect Mol Biol..

[19] Bao YY (2009). Gene expression profiling of resistant and susceptible *Bombyx mori* strains reveals nucleopolyhedrovirus-associated variations in host gene transcript levels. Genomics.

[20] Chen YR (2013). The transcriptome of the baculovirus *Autographa californica* multiple nucleopolyhedrovirus (AcMNPV) in *Trichoplusia ni* cells. J Virol..

[21] Li M (2014). A transcriptome analysis suggests apoptosis-related signaling pathways in hemocytes of *Spodoptera litura* after parasitization by *Microplitis bicoloratus*. PLoS ONE.

[22] Wang XY (2016). Comparative transcriptome analysis of *Bombyx mori* (Lepidoptera) larval midgut response to BmNPV in susceptible and nearIsogenic resistant strains. PLoS ONE.

[23] Yu Q (2016). Transcriptome analysis of the SL221 cells at the early stage during *Spodoptera litura* nucleopolyherovirus infection. PLoS ONE.

[24] Xue J (2012). Dynamic interactions between *Bombyx mori* nucleopolyhedrovirus and its host cells revealed by transcriptome analysis. J Virol..

[25] Bideshi DK, Tang Y, Bigot Y, Federici BA (2005). A viral caspase contributes to modified apoptosis for virus transmission. Genes Dev..

[26] Bideshi DK (2006). Genomic sequence of *Spodoptera frugiperda ascovirus 1a*, an enveloped, double-stranded DNA insect virus that manipulates apoptosis for viral reproduction. J Virol..

[27] Bigot Y (2009). Symbiotic virus at the evolutionary intersection of three types of large DNA viruses; iridoviruses, ascoviruses, and ichnoviruses. PLoS ONE.

[28] Zaghloul H, Hice R, Arensburger P, Federici BA (2017). Transcriptome analysis of the *Spodoptera frugiperda* ascovirus *in vivo* provides insights into how its apoptosis inhibitors and caspase promote increased synthesis of viral vesicles and virion progeny. J Virol..

[29] O’Reilly DR, Brown MR, Miller LK (1992). Alteration of ecdysteroid metabolism due to baculovirus infection of the fall armyworm *Spodoptera frugiperda* host ecdysteroids are conjugated with galactose. Insect Biochem Mol Biol..

[30] O’Reilly DR, Miller LK (1990). Regulation of expression of a baculovirus ecdysteroid UDP glucosyltransferase gene. J Virol..

[31] Chen YR (2014). Transcriptome responses of the host *Trichoplusia ni* to infection by the baculovirus *Autographa californica* multiple nucleopolyhedrovirus. J Virol..

[32] Lyupina YV (2010). An important role of the heat shock response in infected cells for replication of baculoviruses. Virology.

[33] Salem TZ, Zhang F, Xie Y, Thiem SM (2011). Comprehensive analysis of host gene expression in *Autographa californica* nucleopolyhedrovirus infected *Spodoptera frugiperda* cells. Virology.

[34] Lemaitre B, Hoffmann J (2007). The host defense of *Drosophila melanogaster*. Annu Rev Immunol..

[35] Gunaratna RT, Jiang H (2013). A comprehensive analysis of the *Manduca sexta* immunotranscriptome. Dev Comp Immunol..

[36] Meng Q (2015). Transcriptomic insight into the immune defenses in the ghost moth, *Hepialus xiaojinensis*, during an *Ophiocordyceps sinensis* fungal infection. Insect Biochem. Mol Biol..

[37] Tanaka H (2008). A genome wide analysis of genes and gene families involved in innate immunity of *Bombyx mori*. Insect Biochem. Mol Biol..

[38] Waterhouse RM (2007). Evolutionary dynamics of immune-related genes and pathways in disease-vector mosquitoes. Science.

[39] Zou Z (2007). Comparative genomic analysis of the *Tribolium* immune system. Genome Biol..

[40] Dostert C (2005). The Jak-STAT signaling pathway is required but not sufficient for the antiviral response of *Drosophila*. Nat Immunol..

[41] Sa´nchez-Vargas I (2009). Dengue virus type 2 infections of *Aedes aegypti* are modulated by the mosquito’s RNA interference pathway. PLoS Pathog..

[42] Xi Z, Ramirez JL, Dimopoulos G (2008). The *Aedes aegypti* toll pathway controls Dengue virus infection. PLoS Pathog..

[43] Kingsolver MB, Hardy RW (2012). Making connections in insect innate immunity. Proc Natl Acad Sci USA.

[44] Xu Y, Zhou WW, Zhou YJ, Wu JX, Zhou XP (2012). Transcriptome and comparative gene expression analysis of *Sogatella furcifera* (Horvath) in response to Southern rice black-streaked dwarf virus. PLoS ONE.

[45] Emmett SR, Dove B, Mahoney L, Wurm T, Hiscox JA (2005). The cell cycle and virus infection. Methods Mol Biol..

[46] Taniguchi C, Emanuelli B, Kahn C (2006). Critical nodes in signalling pathways: insights into insulin action. Nat Rev Mol Cell Biol..

[47] Ganshan K, Chawla A (2014). Metabolic regulation of immune responses. Annu Rev Immunol..

[48] Seo JY, Cresswell P (2013). Viperin regulates cellular lipid metabolism during human cytomegalovirus infection. PLoS Pathog..

[49] Schoggins JW, Randall G (2013). Lipids in innate antiviral defense. Cell Host Microbe..

[50] Levene P, Meyer G (1912). The action of leucocytes on glucose. J Biol Chem..

[51] Ardawi M, Newsholme E (1982). Maximum activities of some enzymes of glycolysis, the tricarboxylic acid cycle and ketone-body and glutamine utilization pathways in lymphocytes of the rat. Biochem J..

[52] Newsholme E, Crabtree B, Ardawi M (1985). Glutamine metabolism in lymphocytes: its biochemical, physiological and clinical importance. Q J Exp Physiol..

[53] Lester SN, Li K (2014). Toll-like receptors in antiviral innate immunity. J Mol Biol..

[54] Mogensen TH, Paludan SR (2001). Molecular pathways in virus-induced cytokine production. Microbiol Mol Biol Rev..

[55] Cunningham AL (2006). The cycle of human herpes simplex virus infection: virus transport and immune control. J Infect Dis..

[56] Leoni V, Gianni T, Salvioli S, Campadelli-Fiume G (2012). Herpes simplex virus glycoprotein gH/gL and gB bind Toll-like receptor 2, and soluble gH/gL is sufficient to activate NF-κB. J Virol..

[57] Ma Y, He B (2014). Recognition of herpes simplex viruses: Toll-like receptors and beyond. J Mol Biol..

[58] Conesa A (2005). Blast2GO: a universal tool for annotation, visualization and analysis in functional genomics research. Bioinformatics.

[59] Quevillon E (2005). InterProScan: protein domains identifier. Nucleic Acids Res..

[60] Langmead B, Salzberg SL (2012). Fast gapped-read alignment with Bowtie 2. Nat Methods.

[61] Li B, Dewey CN (2011). RSEM: Accurate transcript quantification from RNA-Seq data with or without a reference genome. BMC Bioinformatics.

