# Supplementary material for: Response analysis of host Spodoptera exigua larvae to infection by Heliothis virescens ascovirus 3h (HvAV-3h) via transcriptome
Source: Sci Rep. 2018 Mar 29;8:5367. doi: 10.1038/s41598-018-23715-6 (PMC5876357; doi:10.1038/s41598-018-23715-6)
Supplement: Supplementary file 1 — Supplemental file 1 [file 41598_2018_23715_MOESM1_ESM.pdf]

List of Supplemental Materials

Response analysis of host *Spodoptera exigua* larvae to infection by Heliothis virescens ascovirus 3h (HvAV-3h) via transcriptome

Huan Yu, Zi-Qi Li, Lei He, Yi-Yi Ou-Yang, Ni Li, and Guo-Hua Huang

Supplementary Figures

Figure S1. Venn diagram of *Spodoptera exigua* unigenes annotated with different databases.

Figure S2. Statistics of differentially expressed genes.

Figure S3. Validation of transcriptome data via qPCR.

Figure S4. Correlation analyses between gene expression ratios from qPCR and transcriptome data.

Figure S5. Cluster analyses of DEGs in the HvAV-3h early infection stage.

Figure S6. Cluster analyses of DEGs in the HvAV-3h mass propagation stage and late infectious stage.

Figure S7. The Organismal system in KEGG annotation of host larval DEGs from different comparable groups.

Supplementary Tables

Table S1. Summary of Illumina RNA-seq reads generated from the HvAV-3h infected *Spodoptera exigua* larvae and the number of reads used for de novo assembly.

Table S2. Categorisation and assembly statistics for *S. exigua* larvae transcriptome

Table S3. Species similarity of unigenes BLAST.

Table S4. Annotations of *S. exigua* unigenes.

Table S5. Read counts of *S. exigua* unigenes at various times post infection

Table S6. Average FPKM values of *S. exigua* unigenes at various times post infection

Table S7. Most highly induced unigenes.

Table S8. Most highly up-regulated unigenes.

Table S9. Most highly depressed unigenes.

Table S10. Most highly down-regulated unigenes.

Table S11. *S. exigua* intersection DEGs of different time point vs CK comparisons cluster analyses (Fig 1B).

Table S12. *S. exigua* union DEGs of different time point vs CK comparisons cluster analyses (Fig 1C).

Table S13. Unigenes involved in 6h vs CK cluster analysis (Fig S5A).

Table S14. Unigenes involved in 12h vs CK cluster analysis (Fig S5B).

Table S15. Unigenes involved in 12h vs 6h cluster analysis (Fig S5C).

Table S16. Hypothetical host stress response genes.

Table S17. Intersection DEGs of 6h vs CK and 12h vs CK cluster analysis (Fig S5D).

Table S18. Intersection DEGs of 6h vs CK and 12h vs 6h cluster analysis (Fig S5E).

Table S19. Unigenes involved in 72h vs 12h cluster analysis (Fig S6A).

Table S20. GO annotation of unigenes involved in 72h vs 12h cluster analysis.

Table S21. KEGG annotation of unigenes involved in 72h vs 12h cluster analysis.

Table S22. Hypothetical host genes responded to viral proliferation (Fig 2B).

Table S23. Unigenes involved in 168h vs 72h cluster analysis (Fig S6B).

Table S24. Eight highly up-regulated unigenes in the late infection stage.

Table S25. Hypothetical host response genes in dying struggling stage.

Table S26. GO annotation of all DEGs.

Table S27. GO annotation of DEGs in 6h vs CK.

Table S28. GO annotation of DEGs in 12h vs CK.

Table S29. GO annotation of DEGs in 72h vs CK.

Table S30. GO annotation of DEGs in 168h vs CK.

Table S31. KEGG annotation of all DEGs.

Table S32. KEGG annotation of DEGs in 6h vs CK.

Table S33. KEGG annotation of DEGs in 12h vs CK.

Table S34. KEGG annotation of DEGs in 72h vs CK.

Table S35. KEGG annotation of DEGs in 168h vs CK.

Table S36. DEGs in 72h vs 12h comparison enrichment analysis in Organismal systems of KEGG.

Table S37. Pathways in Metabolism KEGG terms from 6h vs CK DEGs.

Table S38. Pathways in Metabolism KEGG terms from 12h vs CK DEGs.

Table S39. Pathways in Metabolism KEGG terms from 72h vs CK DEGs.

Table S40. Pathways in Metabolism KEGG terms from 168h vs CK DEGs.

Table S41. Most abundant annotated metabolism pathways in KEGG with 6h vs CK DEGs.

Table S42. Most abundant annotated metabolism pathways in KEGG with 12h vs CK DEGs

Table S43. Most abundant annotated metabolism pathways in KEGG with 72h vs CK DEGs

Table S44. Most abundant annotated metabolism pathways in KEGG with 168h vs CK DEGs

Table S45. Primers used in this study
