# Supplementary material for: Response analysis of host Spodoptera exigua larvae to infection by Heliothis virescens ascovirus 3h (HvAV-3h) via transcriptome
Source: Sci Rep. 2018 Mar 29;8:5367. doi: 10.1038/s41598-018-23715-6 (PMC5876357; doi:10.1038/s41598-018-23715-6)
Supplement: Supplementary file 2 — Supplemental file 2 [file 41598_2018_23715_MOESM2_ESM.pdf]

Supplementary Figures

Response analysis of host *Spodoptera exigua* larvae to infection by Heliothis virescens ascovirus 3h (HvAV-3h) via transcriptome

Huan Yu, Zi-Qi Li, Lei He, Yi-Yi Ou-Yang, Ni Li, and Guo-Hua Huang*

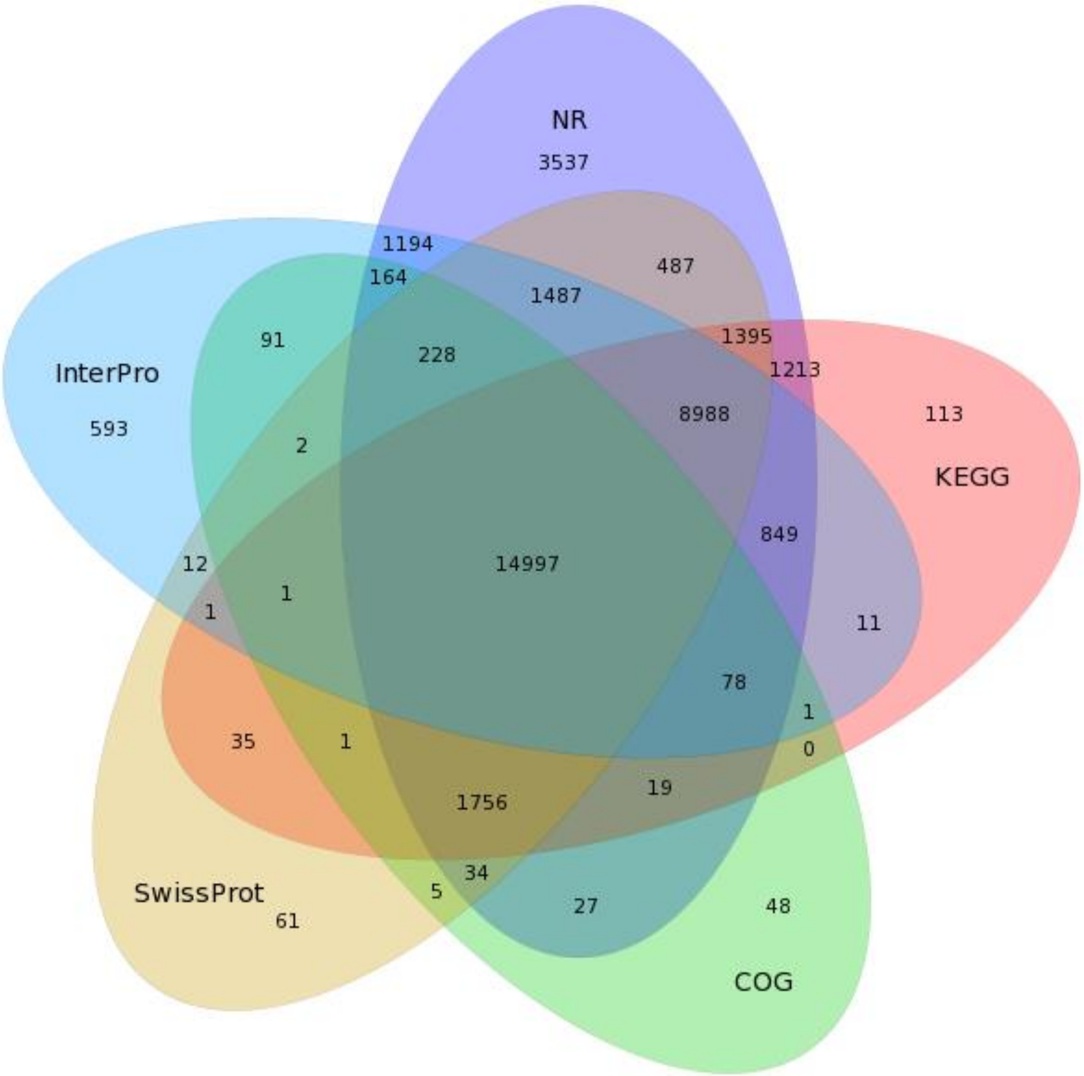

Figure S1 Venn diagram of *Spodoptera exigua* unigenes annotated with different databases. Seven databases were used during annotation, including Nr, Nt, Swissprot, KEGG, COG, Interpro and GO. The Nr, InterPro, SwissProt, COG and KEGG annotated unigene numbers are shown in this figure.

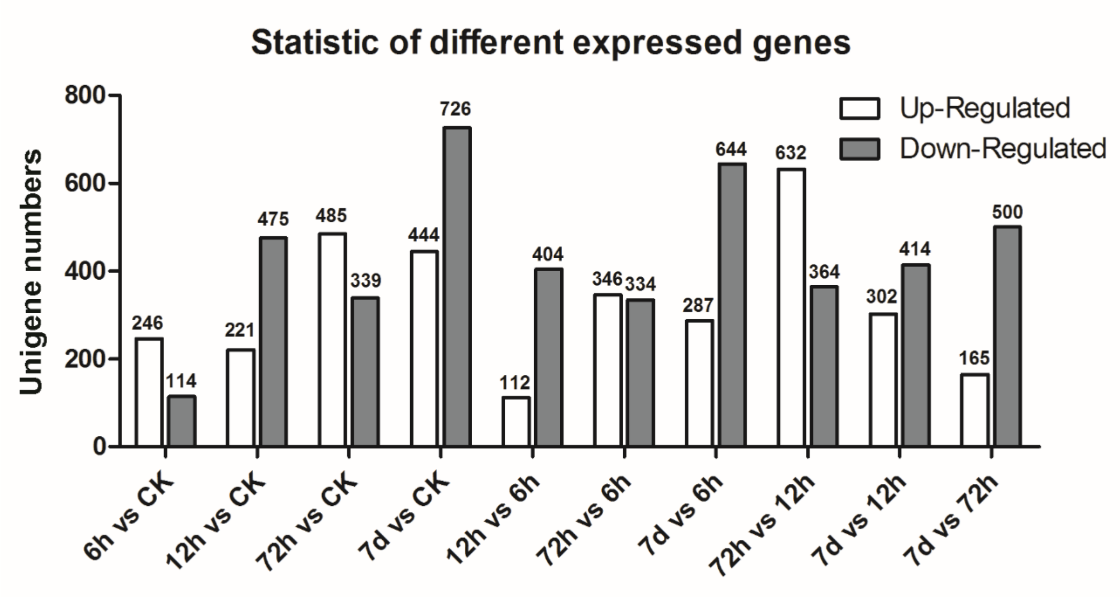

Figure S2 Statistics of differentially expressed genes. Differentially expressed unigenes in different comparisons are illustrated. White columns: Up-regulated unigenes; Gray columns: Down-regulated unigenes.

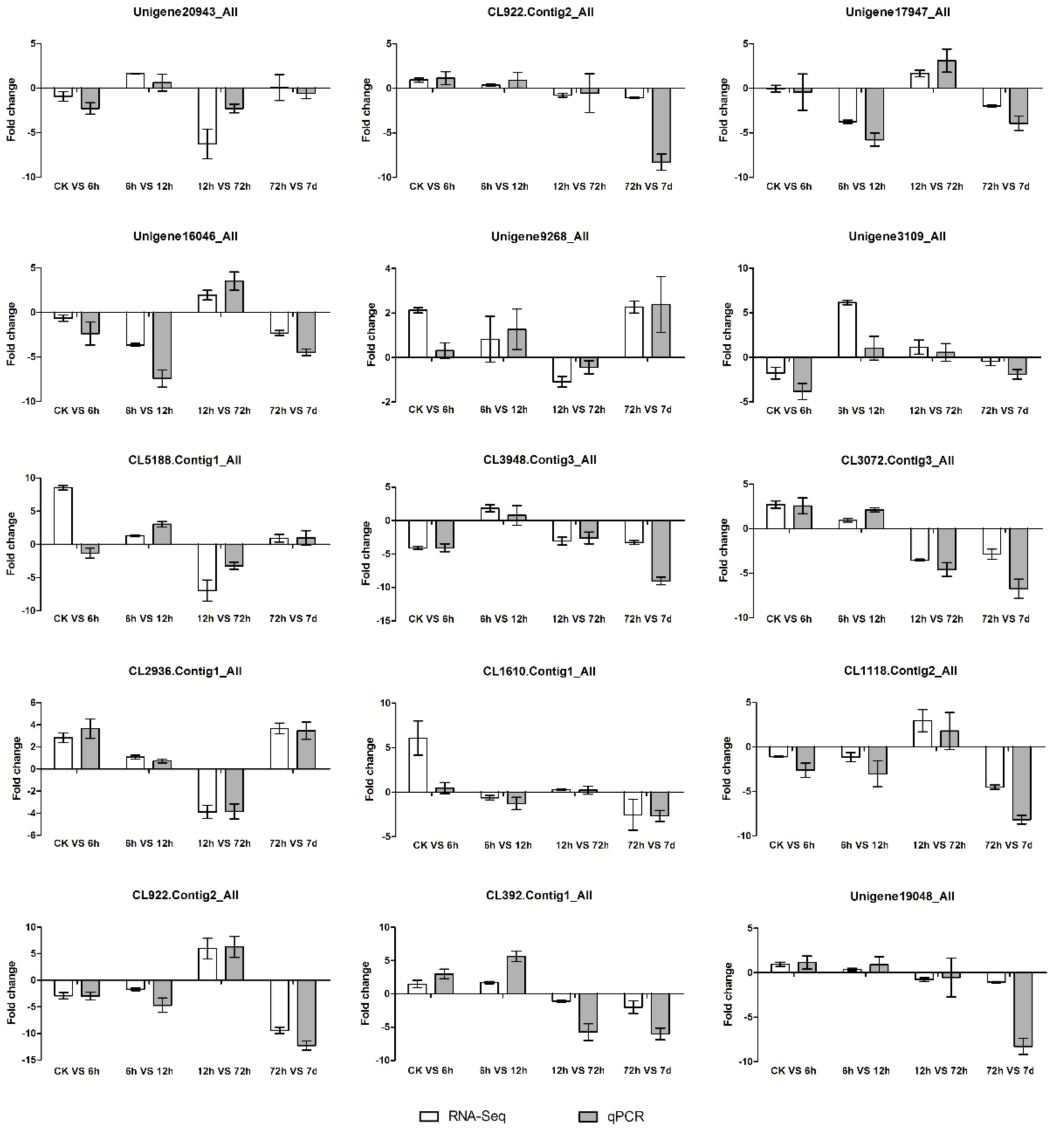

Figure S3 Validation of transcriptome data via qPCR. Fifteen unigenes were randomly selected to investigate the relative expression level by qPCR. The qPCR expression levels were then calculated into Log2Fold change values (gray columns) and compared with specific FPKM Log2Fold change values obtained from transcriptome data (white columns). The error bars stand for standard error.

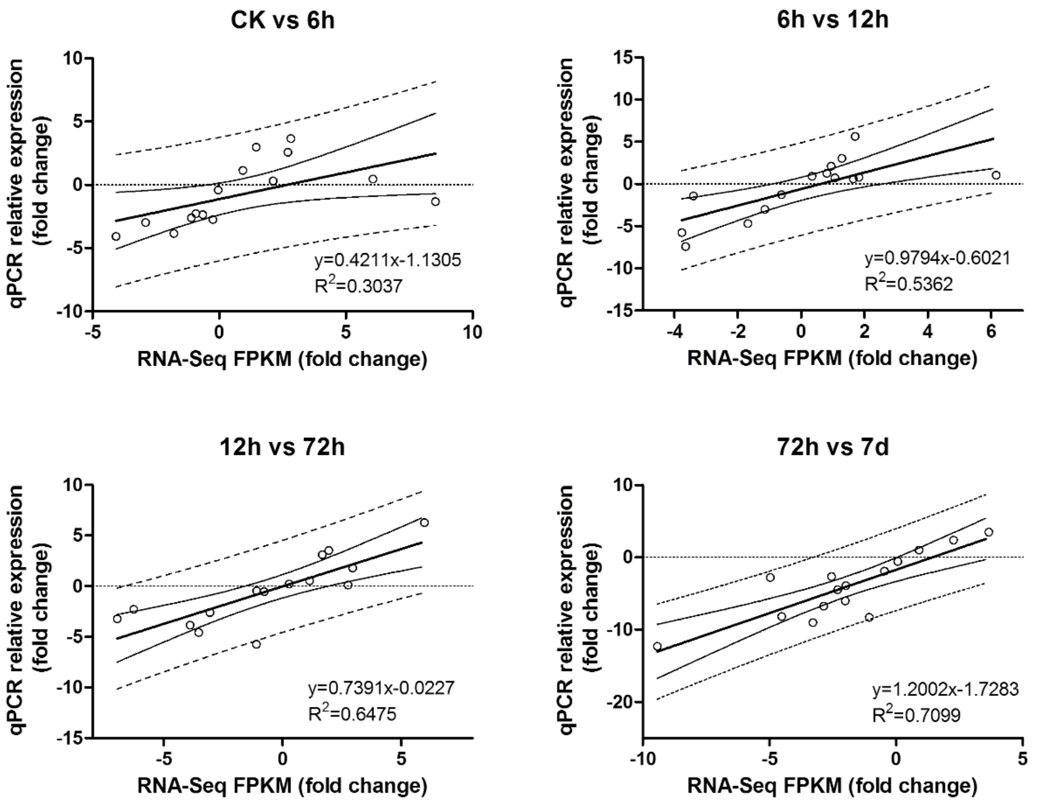

Figure S4 Correlation analyses between gene expressional ratios from qPCR and transcriptome data. Correlation between qPCR and transcriptome data obtained from 6h vs CK (A), 12h vs 6h (B), 72h vs 12h (C) and 168h vs 72h (D) comparisons were performed separately. The middle oblique lines stand for the regression lines, and the regression equations as well as the R square values are shown in the left lower corner in each of the subfigures. The solid curves stand for the 95% confidence intervals of each regression. The dotted curves stand for the 90% confidence intervals of each regression.

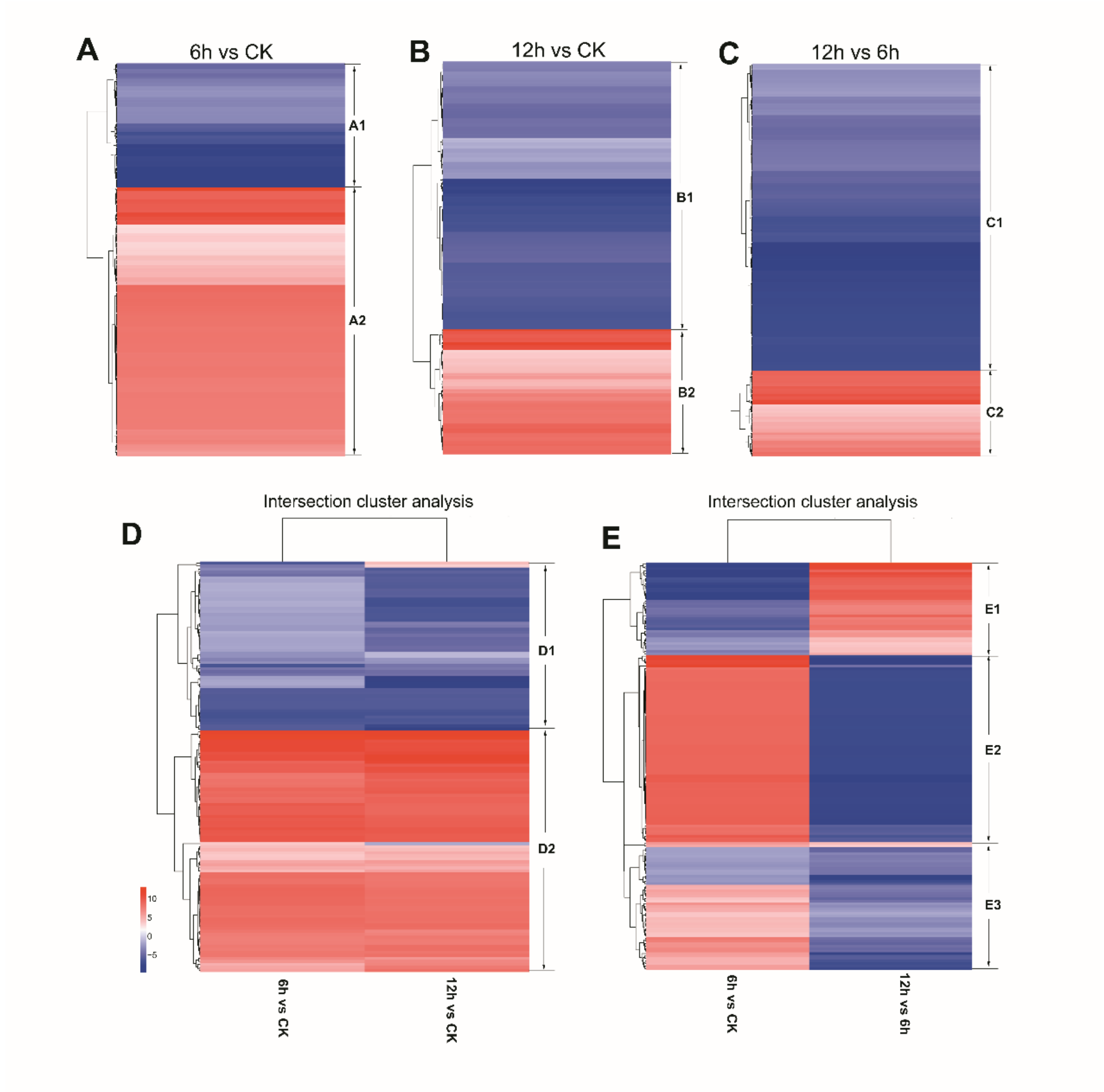

Figure S5 Cluster analyses of DEGs in the HvAV-3h early infection stage. Cluster analyses of DEGs from 6h vs CK (A), 12h vs CK (B) and 12h vs 6h (C) included two major sub-groups in each (labeled with letter and number on the right). The intersection cluster analysis (C) and union cluster analysis (D) of DEGs obtained from 6h vs CK and 12h vs CK included two (D1-D2) and three (E1-E3) major sub-groups, respectively.

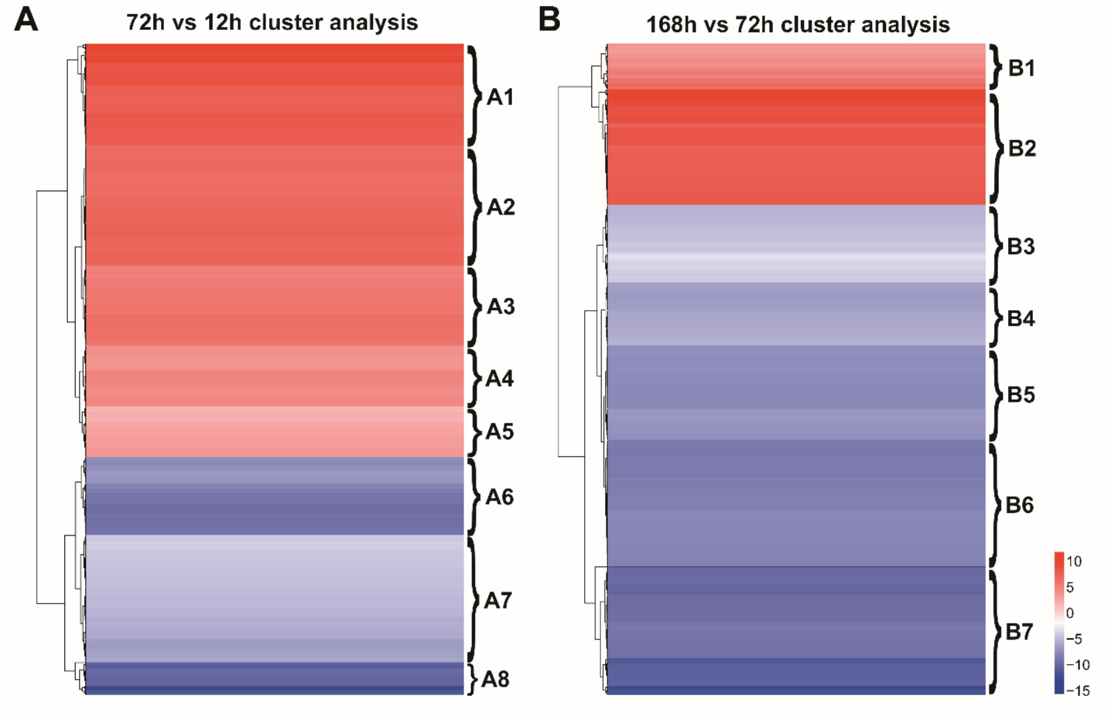

Figure S6 Cluster analyses of DEGs in the HvAV-3h mass propagation stage and late infectious stage. A. Cluster analysis of DEGs obtained from 72h vs 12h, eight major sub-groups are included (A1-A8). B. Cluster analysis of DEGs obtained from 168h vs 72h, seven major sub-groups are included (B1-B7).

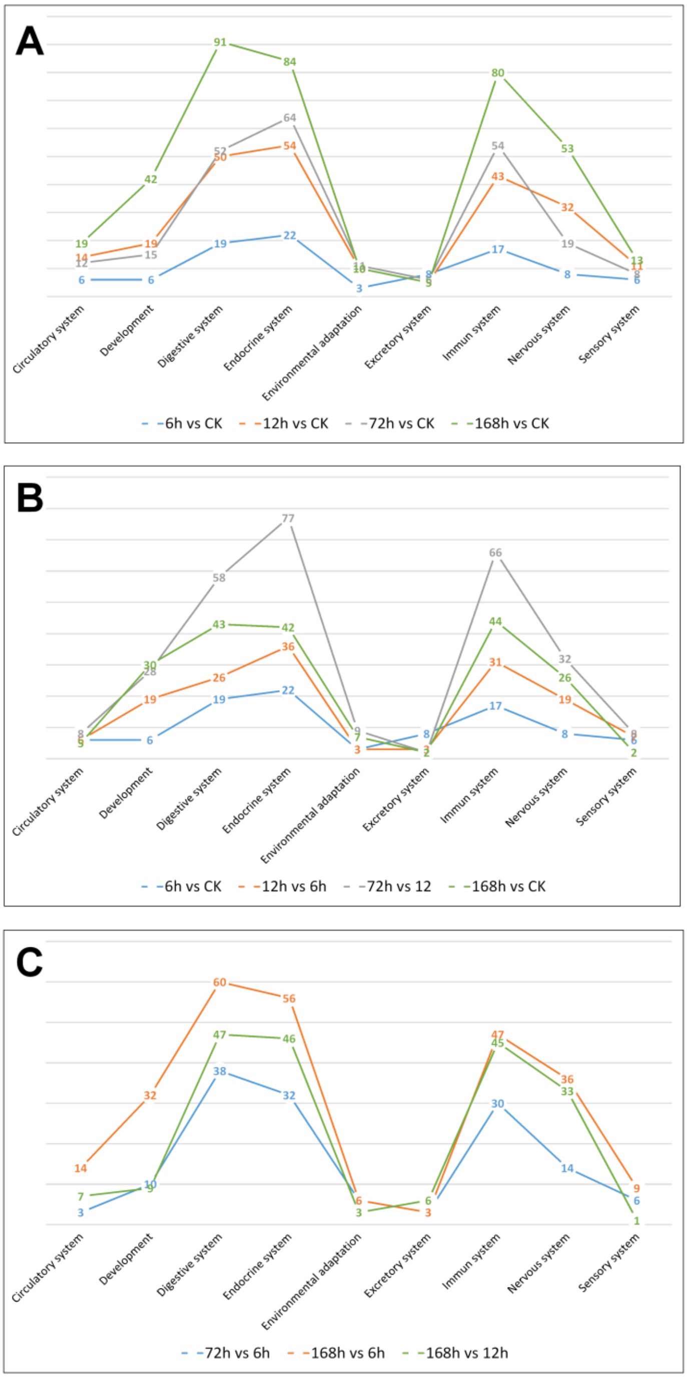

Figure S7 The Organismal system in KEGG annotation of host larval DEGs from different comparable groups.
